# Data Mining of Sediment Microbiomes of the Tibetan Plateau Revealed a Genomic Repository of Ancient Lineages and Adaptive Evolution of Asgardarchaeota

**DOI:** 10.34133/research.1213

**Published:** 2026-03-31

**Authors:** Xiaoke Chen, Nan Wang, Chuanqi Jiang, Shuai Luo, Mingyue Cheng, Dongliang Chu, Che Hu, Peng Zhang, Kai Chen, Fangdian Yang, Jie Xiong, Kang Ning, Wei Miao

**Affiliations:** ^1^ State Key Laboratory of Breeding Biotechnology and Sustainable Aquaculture, Institute of Hydrobiology, Chinese Academy of Sciences, Wuhan, China.; ^2^ Key Laboratory of Molecular Biophysics of the Ministry of Education, Hubei Key Laboratory of Bioinformatics and Molecular-imaging, Center of AI Biology, Department of Bioinformatics and Systems Biology, College of Life Science and Technology, Huazhong University of Science and Technology, Wuhan 430074, Hubei, China.; ^3^ State Key Laboratory of Crop Genetic Improvement and National Centre of Plant Gene Research, Huazhong Agricultural University, Wuhan 430070, China.; ^4^ Laboratory of Tibetan Plateau Wetland and Watershed Ecosystem, College of Science, Tibet University, Lhasa, China.

## Abstract

The extreme climatic conditions of the Tibetan Plateau foster unique microbial communities, especially in the sediment ecosystem. A thorough understanding of these communities could facilitate revealing their microbial diversity, biological resources, and response to climate change. Here, we have constructed the Tibetan Plateau Microbial Catalog of Sediment (TPMC-S) based on 248 metagenomic sediment samples from the Tibetan Plateau. We identified 511,056,752 nonredundant genes and recovered 13,696 metagenome-assembled genomes with enormous phylogenetic novelty (over 90% novel species), far exceeding other contemporary Tibetan microbial catalogs and expanding the microbial functional diversity. We also revealed that similarities of sediment microbial communities followed the distance–decay relationship. Furthermore, sediments contained a high proportion of evolutionarily “possible ancient species (PAS)” compared with paired aquatic samples, especially ancient archaeal lineages, suggesting a microbial “sedimentary archive” in sediment. Finally and most importantly, Asgardarchaeota, including 2 potentially novel genera, were identified from the sediments, and their latest divergence predated the uplift of the Tibetan Plateau, while they still gained functions to adapt to extreme environments. Our findings positioned the Tibetan Plateau as both a genomic repository of microbial antiquity, especially Asgardarchaeota, and an active arena for modern extremophile innovation, providing insights for deciphering microbial resilience strategies in climate-sensitive ecosystems and informing novel bioprospecting efforts.

## Introduction

The Tibetan Plateau, hailed as the “Roof of the World” and the “Third Pole”, boasts a unique topography and climate that give rise to extreme environments characterized by drastic temperature fluctuations, hypoxic atmosphere, and intense ultraviolet (UV) radiation [1–3]. These stressors render the Tibetan Plateau one of the most fragile and sensitive regions to external influence [4,5]. Originating from the Indian-Eurasian collision [~55 to 60 million years ago (Ma)] [6–8], the plateau’s sediments serve as both ecological keystones of past biological processes and active habitats [9–11]. During the long formation process, the plateau’s natural ecology remained largely pristine and undisturbed [12,13], as were its microbial communities, which may preserve ancient, yet undiscovered, microbial lineages that might have deep phylogenetic origins. However, accelerating anthropogenic disturbances and climate change have imposed irreversible impacts on these microbial lineages [5,14], necessitating urgent conservation efforts for these ecologically invaluable yet understudied communities.

Microbial communities on a global scale have undergone extensive exploration, covering diverse ecological environments [15–18]. Groundbreaking initiatives such as the Earth Microbiome Project have shed light on the global diversity and dynamics of microbial life [16,17,19]. On the Tibetan Plateau, previous extensive research has characterized microbiological communities in diverse soil environments [5,20–22] and cataloged microbial life along with biosynthetic gene clusters (BGCs) in aquatic systems [2,9,23–25]. In particular, the plateau’s expansive geography sources major river systems, forming diverse sedimentary environments [11,26,27] that are presumed to harbor rich microbial assemblages derived from upstream sources [28,29]. However, a systematic, genome-resolved understanding of the microbial landscape across these diverse sediment ecosystems—spanning different latitudes, altitudes, and habitat types—remains elusive, hindering our ability to investigate the evolutionary timescales of sediment microbial lineages and the distribution and adaptive mechanisms of key taxa to specific environmental niches.

The extreme environments usually harbor a unique reservoir of microbial phylogenetic diversity, comprising lineages that have undergone long-term evolution under persistent stressors (e.g., low temperature, high UV, and salinity) [30–32]. This unique pool includes “possible ancient species (PAS)” whose differentiation could be tracked back millions or even tens of millions of years ago, which hold important scientific value for reconstructing Earth’s evolutionary history [33–35]. The sedimentary layers of the Tibetan Plateau not only function as a natural repository, preserving microbial fossils that have remained entombed for eons, but also serve as dynamic arenas where evolutionary processes, such as genetic adaptation and niche specialization, can be investigated [36–38]. Understanding the genetic underpinnings of microbial resilience in these sediments provides crucial insights into how life adapts to and persists under extreme environmental conditions [34,39]. Despite the importance of these discoveries, the evolutionary trajectories and the specific adaptive mechanisms that enable microbial lineages, particularly those with deep evolutionary origins, to thrive in the plateau’s contemporary extreme environments remain largely unresolved, as well as the explicit coupling mechanisms linking the functional genes (e.g., for biosynthesis and stress response) to measured environmental gradients remain unexplored, resulting in a critical knowledge gap that limits our ability to decipher the principles of microbial adaptation and to predict ecosystem responses to ongoing environmental change.

To address these issues, we have established the Tibetan Plateau Microbial Catalog of Sediment (TPMC-S) through large-scale metagenomic analysis of the Tibetan Plateau sediments, revealing microbial diversity across extreme plateau ecosystems. In addition, we unveiled the distance–decay relationship (DDR) governing microbial biogeography across spatial-temporal gradients. Besides, divergence trajectories analysis was conducted to assess the role of sediment as a “sedimentary archive” containing ancient microbes as well as their functional genes. We also recovered Asgardarchaeota metagenome-assembled genomes (MAGs) from the sediments, including 2 potentially novel genera, and further revealed their phylogenetic divergence predated regional geological uplift events. Functional analyses further revealed that these extremophilic Asgardarchaeota have developed specialized functional differentiations, including their adaptive mechanisms for osmoregulation and cryoprotection. Collectively, this work elucidates the evolutionary trajectory and ecological plasticity of Asgardarchaeota in extreme ecological environments, supports the role of the Tibetan Plateau’s sediments as a reservoir of ancestral microbial lineages, and provides critical genomic resources for understanding microbial resilience mechanisms and informing biogeochemical engineering strategies in climate-vulnerable ecosystems.

## Results

### Microbial community samples from the Tibetan Plateau sediments

**Fig. 1.**
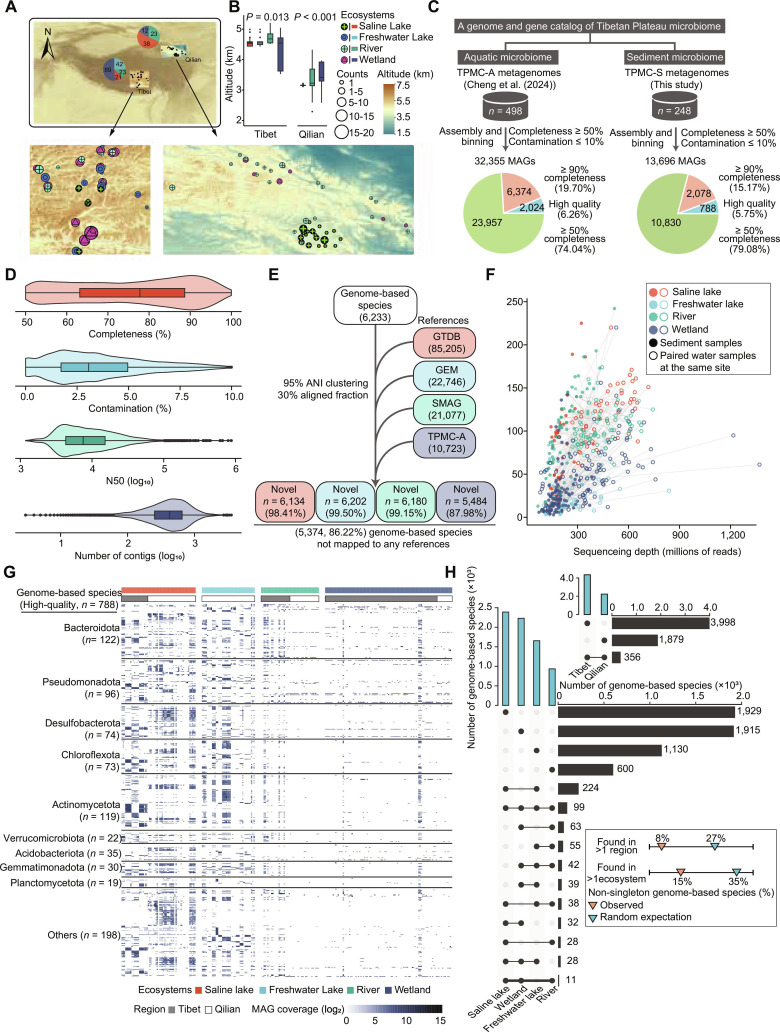
Quality summary of metagenome-assembled genomes (MAGs) and their species-level clustering, distribution, and coverage. (A) Geographic distribution of metagenomic samples from 4 sediment ecosystems (Freshwater Lakes, Rivers, Saline Lakes, and Wetlands) in 2 large-scale regions (Tibet and Qilian). Specific distribution of metagenomic samples in Tibet and Qilian is shown. The pie chart shows the number of samples collected at different sample sites. (B) Box plot of elevation in Tibet and Qilian (Tibet Kruskal–Wallis test: *P* = 0.0131; Qilian Kruskal–Wallis test: *P* < 0.001). Whiskers denote the lowest and highest values within the 1.5× interquartile range from the first and third quartiles, respectively. (C) TPMC-S contains a total of 13,696 MAGs recovered from 248 sediment metagenomes, 198 of which have paired aquatic metagenomes in TPMC-A. (D) Distribution of quality metrics for the TPMC-S MAGs (*n* = 13,696). Boxes represent the interquartile range between the first and third quartiles, and the line inside represents the median. Whiskers denote the lowest and highest values within the 1.5× interquartile range from the first and third quartiles, respectively. (E) TPMC-S MAGs are clustered into 6,233 genome-resolved species. Representative genomes from GTDB R214 (*n* = 85,205), GEM (*n* = 22,746), SMAG (*n* = 21,077), and TPMC-A (*n* = 10,723) are included in the clustering to identify the novelty of the TPMC-S species. (F) Number of recovered MAGs in samples with different sequencing depths. Paired samples are connected by gray lines. (G) Distributions of the TPMC-S species across diverse ecosystems and regions. The majority of TPMC-S species with more than one MAG is restricted to individual aquatic ecosystems and regions. (H) Coverage of high-quality TPMC-S species in all the samples.

To explore the microbial genomic resources in the sediments of the Tibetan Plateau, we assembled a comprehensive dataset comprising 248 sediment metagenomic samples. These samples were collected from various ecosystems across 2 primary regions: the central Tibetan Plateau (abbreviated as Tibet, *n* = 175), including River (*n* = 23), Saline Lake (*n* = 21), Wetland (*n* = 89), and Freshwater Lake (*n* = 42), as well as the northeastern margin of the Tibetan Plateau (Qilian Mountains–Qinghai Lake, abbreviated as Qilian, *n* = 73), covering River (*n* = 23), Saline Lake (*n* = 38), and Wetland (*n* = 12). All samples collectively represented 4 distinct ecosystems encompassing River (*n* = 46), Saline Lake (*n* = 59), Wetland (*n* = 101), and Freshwater Lake (*n* = 42) (Fig. 1A and B and Tables S1 and S2).

We have also incorporated publicly available metagenomic data from 243 aquatic samples from our previous study on the Tibetan Plateau [23], sourced from identical collection sites but varying in depth (Table S2; details in Methods) for comparison. These aquatic samples were strategically selected to represent the dominant, long-standing aquatic ecosystems of the plateau. Their formation is closely linked to the region’s geological history, with many water bodies having evolved over tens of thousands to millions of years. The water bodies and the sediments beneath the surface water bodies are the cumulative products of the long-term environmental processes and microbial community succession and preservation. We have defined “Paired aquatic and sediment samples” as those obtained from the same sites. This unique dataset enables a systematic comparison of the microbiomes between aquatic and sediment environments.

### TPMC-S provided a vast and unique genomic resource including over 5,000 novel MAGs

Using a standardized cataloging pipeline consistent with that employed for the Tibetan Plateau aquatic microbiomes (TPMC-A), we reliably reconstructed 13,696 (96.48%) refined MAGs with a mean completeness of 75.9% and a mean contamination rate of 3.5% (Fig. 1C and D and Table S3). Among these MAGs, 9,420 (68.8%) achieved a quality score above 50 [defined as completeness rate – (5 × contamination rate)], and 788 (5.75%) were assigned as high-quality genomes, featuring the presence of the 23S, 16S, and 5S ribosomal RNA (rRNA) genes and at least 18 transfer RNAs (tRNAs) (Table S3). Analysis of MAG characteristics, including assembly size and guanine and cytosine (GC) content, revealed significant differences across ecosystems (*P* < 2.2 × 10^–16^) but not between regions (*P* > 0.1; Table S4). Specifically, MAGs recovered from Saline Lakes sediment exhibited the highest completeness (median = 80.7%) and assembly size (median = 2.7 Mb), while those from Wetlands showed the lowest values (Table S3).

Comparative analysis revealed that TPMC-S contained a large number of novel genomes. We clustered 13,696 sediment MAGs into 6,233 genome-resolved species using a whole-genome average nucleotide identity (ANI) threshold of 95% and an aligned fraction (AF) threshold of 30% (Fig. 1E). To assess the novelty of these representative TPMC-S species, we compared them with representative genomes from the Genome Taxonomy Database (GTDB release R214) [40], the Earth’s Microbiomes catalog (GEM) [41], the Global Soil Genomes (SMAG database) [42], and the Tibetan Plateau Microbial Catalog (TPMC-A) [23], and 98.41%, 99.50%, 99.15%, and 87.98% of TPMC-S species had low sequence identity with these reference genomes, respectively (Table S5). Remarkably, 5,374 species (86.22%) were unique to TPMC-S, further highlighting the unique microbial genomic reservoirs of Tibetan Plateau sediments. This speciation was likely due to ecosystem isolation of the Tibetan Plateau and natural selection under the influence of extreme environmental conditions.

Interestingly, the number of recovered MAGs of sediment samples consistently surpassed that of aquatic samples collected at the same sampling site (Fig. 1F). Although metagenomic sequencing depth approached saturation for both sediment and aquatic samples, neither was fully saturated (Fig. 1F). Sediment samples exhibited steeper saturation curves for MAG recovery and gene identification compared to aquatic samples, indicating that sediments contained more diversity in both genome recovery and functional genes at the same sequencing depth (Fig. S1). This suggested that sediment ecosystems possessed richer microbial diversity and functional potential compared to aquatic environments.

We also investigated the species distribution across Tibetan Plateau sediment ecosystems and discovered that different sediment ecosystems had unique sets of microbial species, respectively. Most genome-resolved species (with at least one MAG support) were exclusive to a single region (*n* = 5,877, 94.29%) or ecosystem (*n* = 5,574, 89.43%) (Fig. 1G). Notably, sediment species from Saline Lakes (*n* = 1,929, 30.95%) and Wetlands (*n* = 1,915, 30.72%) exhibited the highest recovery rates. Furthermore, non-singleton sediment species (*n* = 4,346), which contained more than one MAG, were largely site-specific (*n* = 3,990, 91.81%) and ecosystem-specific (*n* = 3,687, 84.84%) (Fig. 1G and Table S3). High-quality MAGs (*n* = 788) were predominantly derived from Saline Lakes and Freshwater Lakes (Fig. 1H). These findings underscored the ecosystem-dependent discovery potential of TPMC-S, particularly in Saline Lake ecosystems.

**Fig. 2.**
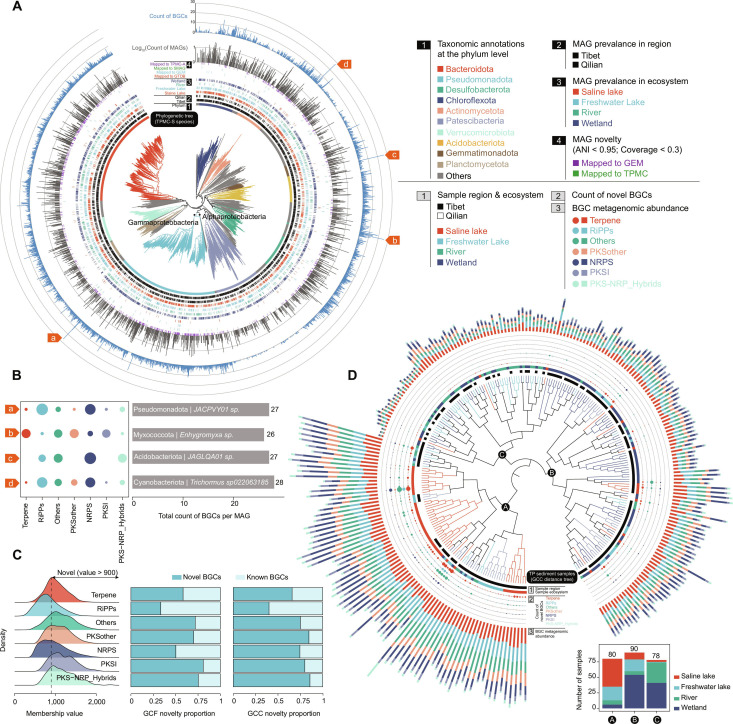
Phylogenetic tree of TPMC-S species and biosynthetic potential across samples. (A) A phylogenetic tree of the TPMC-S genome-resolved species (*n* = 6,233) is constructed based on a concatenated alignment of 120 universally distributed bacterial single-copy genes and the placement of each species in the GTDB-Tk reference tree. Two classes of the phylum Pseudomonadota, Gammaproteobacteria and Alphaproteobacteria, are marked in the tree. The color of the branches indicates the phylum of the species. The outer layers indicate, for each species, the region and ecosystem labels based on its MAGs, the count of MAGs, the largest count of biosynthetic gene clusters (BGCs) among its MAGs, and the novelty (compared to the representative genomes of GTDB, GEM, SMAG, and TPMC-A catalogs, respectively). (B) Species with predicted BGCs > 25 are displayed. The size of the circle corresponds to the proportion of each category of the BGCs in a species. (C) Novelty of each category of the BGCs is compared and displayed by density plots. BGCs with a membership value > 900 against the BiG-FAM reference database are designated as novel. The novelty of gene cluster families (GCFs) and gene cluster clans (GCCs) is determined by the proportion of BGCs they contain. (D) A dendrogram is constructed using Ward clustering based on the Bray–Curtis distance of metagenomic abundances of GCCs across samples. Three clusters are labeled with letters. The numbers of samples for each ecosystem in each cluster are displayed by stacked plots. The color of the branches indicates the regions and ecosystems of the samples. The outer layers indicate, for each sample, the count of novel BGCs and the metagenomic abundances of each category of the BGCs.

Based on comprehensive taxonomic annotations from GTDB, we successfully annotated MAGs of TPMC-S into known taxonomic levels. These MAGs were assigned to 97 known phyla, 220 known classes, 528 known orders, 1,002 known families, 1,586 known genera, and 127 known species across archaea and bacteria (Table S3). We then constructed a phylogeny of the 6,017 bacterial genome-resolved species using 120 concatenated bacterial marker genes and highlighted the top 10 phyla (Fig. 2A). The MAG catalog was dominated by Bacteroidota (*n* = 2,689, 19.63%), Pseudomonadota (*n* = 2,597, 18.96%), Desulfobacterota (*n* = 1,039, 7.59%), and Chloroflexota (*n* = 923, 6.74%) (Fig. S2). Additionally, we recovered 216 archaeal genome-resolved species, dominated by phyla Thermoplasmatota (*n* = 102, 0.74%), Nanoarchaeota (*n* = 77, 0.56%), Halobacteriota (*n* = 63, 0.46%), and Thermoproteota (*n* = 57, 0.42%) (Table S3). Notably, 97.58% of genome-resolved species lacked GTDB representatives, with the majority associated with Bacteroidota (*n* = 1,080, 17.8%), Pseudomonadota (*n* = 1,073, 17.6%), and Patescibacteria (*n* = 490, 8.1%) (Table S5). These “unknown taxa” formed deep-branching, monophyletic clades within their respective phyla in our phylogenetic tree (Fig. 2A), suggesting that they may represent novel families or even higher-order ranks. Their prevalence, especially in understudied phyla like Patescibobacteria, underscores that the Tibetan Plateau sediments are a reservoir for microbial lineages that are phylogenetically distinct from previously sequenced ecosystems. Our catalog has expanded such novel microbial lineages.

### TPMC-S encodes unique functions with over 500 million nonredundant genes

Besides cataloging genomic resources, we extensively explored and cataloged the functional potential within the TPMC-S. We predicted and clustered 701,528,671 open reading frames (ORFs) into 511,056,752 nonredundant gene clusters, referred to as nonredundant genes, which far exceeded the Tibetan Glacier Genome and Gene (TG2G) catalog [2]. The nonredundant gene catalog underwent taxonomic and functional annotations using the Non-Redundant Protein (NR) and Swiss-Prot databases [43], resulting in annotations for 82.98% and 38.26%, respectively (Fig. S3). Further functional annotations using COG, KEGG, CAZy, GO, CARD, and VFDB databases lead to annotations for 78.03%, 44.85%, 1.47%, 5.39%, 0.01%, and 8.23%, respectively [44–48] (Fig. S3). Notably, 47,973,982 (9.39%) nonredundant genes remained unannotated by any of the databases, thereby designating them as novel genes. These new gene resources revealed the remarkable novelty of the ecological functions of TPMC-S.

**Fig. 3.**
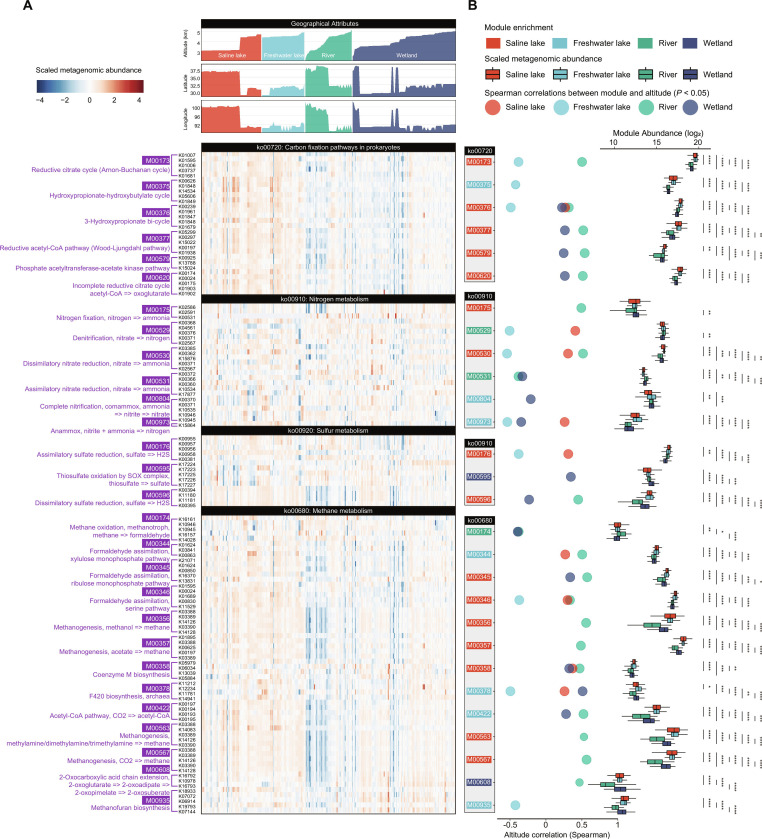
Functional potential of TPMC-S in carbon, nitrogen, and sulfur metabolism. (A) The heatmap shows the metagenome abundances of genes related to carbon fixation and methane, nitrogen, and sulfur metabolism across samples of various ecosystems. The top panel shows the geographical attributes of these samples, including latitude, longitude, and altitude. (B) The boxplots show the differences in metagenomic abundances of the functional modules across ecosystems. The statistical significance is calculated by the 2-sided Mann–Whitney–Wilcoxon test. Boxes represent the interquartile range between the first and third quartiles, and the line inside represents the median. Whiskers denote the lowest and highest values within the 1.5× interquartile range from the first and third quartiles, respectively. The circles represent the significant Spearman correlations (*P* < 0.05) between the modules and the altitude.

Furthermore, the comparative study of functional genes between paired aquatic and sediment samples revealed the functional variations across ecosystems. TPMC-S was enriched in genes associated with carbon, nitrogen, and sulfur cycling (64%), while TPMC-A showed a higher abundance of genes involved in biosynthesis (69%) and antibiotic resistance (Fig. 3A and Figs. S1 and S4). When examining the metagenomic abundances across different ecosystems, significant differences were observed in the genetic components responsible for carbon fixation (*P* < 0.05), as well as methane, nitrogen, and sulfur metabolism (*P* < 0.05) (Fig. 3B). Some modules related to carbon metabolism (e.g., M00173 and M0037) and methane metabolism (e.g., M00346 and M00356) have significantly higher abundances in Saline Lake and Freshwater than in River and Wetland (Fig. S5). Overall, there were numerous and significantly enriched modules related to diverse metabolic pathways (including carbon fixation, methane, nitrogen, and sulfur metabolism), and the main enriched modules were those related to membrane metabolism (Fig. 3B). There were general correlations between these modules and altitude, suggesting the impact of environmental factors on the functions of microbial communities. These findings highlighted the ecological differentiation of microbial communities, with environmental conditions shaping specific metabolic pathways. Moreover, our findings revealed a limited overlap of genes (ranging from 2% to 5%) between sediment and aquatic samples (Fig. S4), indicating strong environmental specificity in each habitat. These outcomes collectively suggested that microbes adjusted their metabolic functions to adapt to their corresponding habitats.

The TPMC-S genes also showed a remarkable functional potential for secondary metabolites. We predicted a total of 26,360 BGCs, which surpassed TG2G [2] and were subsequently categorized into 8 distinct groups (Table S6). Among these, Terpenes (*n* = 7,441, 28.23%) and posttranslationally modified peptide (RiPP, *n* = 6,037, 22.90%) clusters were the most prevalent BGCs (Fig. 2C). Approximately half of the BGCs (*n* = 12,570, 47.69%) were predicted from phyla Pseudomonadota (*n* = 5,088, 19.30%), Bacteroidota (*n* = 5,046, 19.14%), and Desulfobacterota (*n* = 2,436, 9.24%) (Fig. 2A). Moreover, the Saline Lakes in Qilian (*n* = 7,152, 27.13%) and Tibet (*n* = 5,369, 20.37%), along with the Freshwater Lakes (*n* = 6,507, 24.69%) in Qilian, shared the greatest number of predicted BGCs (Table S7). The identified 26,360 BGCs were further clustered into 10,888 gene cluster families (GCFs) and 453 gene cluster classes (GCCs), and compared against the BiG-SLICE database [49] (Table S8). A GCF or GCC was considered novel if less than 20% or 40% of its constituent BGCs were mapped to BiG-SLICE, respectively (see details in Methods; Table S8). Our results show that more than half of the GCFs (*n* = 6,108, 56.10%, Table S9) and GCCs (*n* = 297, 65.56%, Table S10) were novel across the majority of the BGC categories (Fig. 2C). Notably, some GCCs belonged to Terpenes, involving GCC 14707, GCC 18777, and GCC 1962, whose abundance increased with the increase of altitude and regulated the functions of antioxidant, antibacterial, and cell-protection (Fig. S5). The microbes might resist the oxidative stress caused by high UV radiation and low temperatures through these biological synthesis potentials. These findings suggested that Tibetan Plateau microbes might be capable of synthesizing various secondary metabolites with unique structures and functions to cope with harsh environmental conditions.

We next identified 4 BGC-rich species, each from a distinct phylum: Cyanobacteriota, Acidobacteriota, Myxococcota, and Pseudomonadota, each of which contained at least one MAG with over 25 BGCs (Fig. 2B and Table S11), as well as found that their BGCs were enriched in RiPPs and nonribosomal peptide synthase (NRPS). Notably, 3 of them represented newly identified MAGs. Moreover, the discovery of these BGC-rich species across diverse ecosystems suggested their capacity to thrive in various ecological niches. This broad distribution underscored their adaptability and potential ecological significance, which may be crucial for unraveling the mechanisms underlying microbial resilience in extreme environments. These BGC-rich species emerged as promising candidates for investigating the fundamental processes by which microbes adapted to the harsh conditions of the Tibetan Plateau.

Moreover, we investigated the diversity of the biosynthetic potential across diverse sediment ecosystems. Using Ward clustering based on the Bray–Curtis distance of metagenomic abundances of GCCs across all 248 sediment samples, we identified 3 sample clusters: Cluster A consisted mainly of the Saline Lake and Freshwater Lake samples; cluster B mainly consisted of Freshwater Lake and Wetland samples; cluster C mainly consisted of River and Wetland samples (Fig. 2D). Notably, samples within cluster A exhibited the highest abundance of BGCs and the greatest potential for discovering novel BGCs, which was largely attributed to the fact that cluster A was primarily composed of samples from Saline Lakes and Freshwater Lakes. The BGCs with the largest number in cluster A is Terpenes and RiPPs, indicating their roles in the process of environmental adaptation (Fig. S6). These results revealed the unique BGC distribution patterns across diverse ecosystems, suggesting their specific ecological adaptations and interactions within their respective habitats.

### The microbial community compositions and functions followed the DDR

Comparative analysis of microbial communities revealed striking differences in community structure among 4 sediment ecosystems. Saline Lake sediments exhibited the highest microbial alpha-diversity, significantly surpassing freshwater lakes, rivers, and wetlands (*P* < 0.05) (Fig. S7). Notably, wetland communities demonstrated pronounced dominance patterns, with the top 10 phyla accounting for more than 80% of total abundance (Fig. S8). Bray–Curtis distances confirmed distinct beta-diversity patterns across ecosystems (*P* < 0.001) (Fig. S7), although Actinomycetota phylum maintained stable abundances regardless of ecosystem type (*P* > 0.05), suggesting broad environmental adaptability (Fig. S8).

The neutral community model (NCM) revealed the differentiated construction mechanisms of different ecosystems (Fig. S9A). Freshwater Lake (*R*^2^ = −0.255) and Saline Lake (*R*^2^ = −0.128) communities significantly deviated from NCM, while Wetland (*R*^2^ = 0.532) and River (*R*^2^ = 0.483) communities showed better model fit despite extremely low estimated migration rates (*m* < 0.001).

DDR analysis provided complementary evidence for spatial constraints on microbial dispersal. We performed DDR analysis on the sediment samples to explore the effect of distance on microbial community similarity. The results indicated that, in terms of geographical distance, the Bray–Curtis similarity of microbial communities from both Tibet and Qilian decreased as the geographical distance increased (Fig. S9B). A similar trend was observed when considering vertical distance. This distribution pattern was consistent with the microbial genes present in samples from both Tibet and Qilian, respectively (Fig. S9C). These results underscore the spatial heterogeneity in microbial communities and functions within sediment samples, highlighting geographical and vertical distances as pivotal factors influencing microbial similarity.

### Divergence trajectories revealed the prevalent presence of PAS in the sediment

**Fig. 4.**
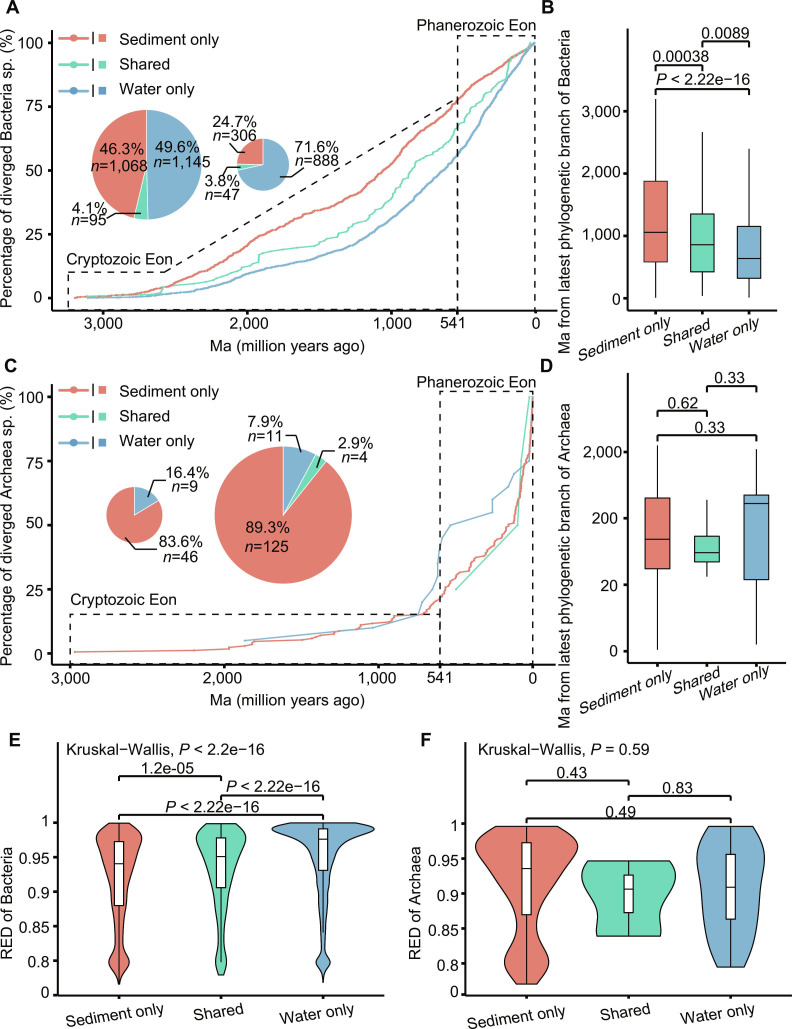
Assessment of the conservation of evolutionarily “possible ancient species (PAS)”. According to the latest divergence time of these species, we classified these species as either PAS (latest divergence time in the Cryptozoic Eon) and “extant species” (latest divergence time in the Phanerozoic Eon). (A and C) Divergence time of “ancient” and “extant” Bacteria and Archaea. The genome-resolved species were categorized into 3 groups: Sediment only (red), Aquatic only (blue), and Shared (green). The line plot shows the proportion of species within each group over time, based on the estimated age of their most recent phylogenetic branches. The dashed line at 600 million years marks the boundary between the Cryptozoic Eon and the Phanerozoic Eon. The inset pie charts depict the proportion of species that belonged to Cryptozoic (left) and Phanerozoic (right) Eons of each group. (A) Bacteria. (C) Archaea. (B and D) The box plots show the average Ma (million years ago) from the latest phylogenetic branches among 3 groups. (B) Bacteria. (D) Archaea. Statistical significance was determined using a 2-sided Mann–Whitney–Wilcoxon test. Boxes represent the interquartile range (IQR) between the first and third quartiles, with the median indicated by the line inside each box. Whiskers extend to the lowest and highest values within 1.5× IQR from the first and third quartiles. (E and F) The violin plots show the differences in relative evolution distance among the 3 groups. (E) Bacteria. (F) Archaea. Statistical significance was determined using Kruskal–Wallis test. ****P < 0.001.

We further analyzed the divergence time of the species in the paired sediment and aquatic ecosystems. Specifically, we estimated the divergence times for an archaeal phylogeny comprising 195 genome-resolved species and a bacterial phylogeny encompassing 3,549 genome-resolved species (Fig. 4; see details in Methods). The results elucidated the evolutionary trajectories of 3,744 species, spanning from the earliest of 3,195.8 Ma for UBA5614 to 8.9 Ma for *Fen-1088* (Fig. 4A and C). These species were further categorized into 3 distinct groups: Sediment only (exclusively in sediment), Water only (exclusively in aquatic), and Shared (present in both environments). The extensive evolutionary timeline was partitioned into the Cryptozoic Eon (prior to 541 Ma) and the Phanerozoic Eon (541 Ma to the present). According to the latest divergence time of these species, we classified these species as either PAS (latest divergence time in the Cryptozoic Eon) or “extant species” (latest divergence time in the Phanerozoic Eon).

Our results revealed that the sediment contained a higher proportion of ancient bacterial species compared to aquatic environments. Within the Cryptozoic Eon, we identified 2,308 ancient bacterial species, with 46.3% exclusive to sediment, 49.6% exclusive to water, and 4.1% shared between both environments (Fig. 4A and Fig. S10). Conversely, the Phanerozoic Eon contained 1,241 extant bacterial species, of which only 24.7% were exclusive to sediment. Notably, throughout the Cryptozoic Eon, the proportion of diverged bacteria from sediment consistently surpassed that from water. Additionally, the average divergence time from the latest phylogenetic branches in sediment was significantly higher than that in water (Fig. 4B) (*P* < 0.01), while the relative evolution distance (RED) of bacteria in sediment was significantly lower (Fig. 4F) (*P* < 0.01). These observations affirmed that the sediment of the Tibetan Plateau contained a higher proportion of PAS, suggesting its role as a “sedimentary archive” for microbial evolution—a geological reservoir that has more effectively preserved ancient microbial lineages and their genomic signatures over long timescales, compared to the adjacent, more dynamic aquatic environments.

A similar trend was more pronounced in Archaea. The PAS exclusive to sediment constituted over 80% of the total (46/55) in the Cryptozoic Eon, while only 9 PAS belonged to water only group (Fig. 4C). Analogously, at the inception of the Phanerozoic Eon, the sediment contained 89.3% (125/140) of extant species, while water only contained 11 species (7.9%). No significant differences were observed between sediment and water for Archaea when we observed the average divergence time from the latest phylogenetic branches or the relative evolutionary distance (Fig. 4D and G). Therefore, it could be inferred that the sediment contained a higher proportion of ancient archaea. All of these results have shown that species enriched in sediment were highly likely to be ancient, indicating that sediment might serve as the “sedimentary archive” for microbes in the Tibetan Plateau.

Collectively, these results indicated that the sediment of the Tibetan Plateau was a treasure trove of microbial resources including PAS, especially ancient archaeal lineages. The conservation of this resource was paramount for preserving microbial diversity in extreme environments.

### The Tibetan Plateau’s sediment was a reservoir of Asgardarchaeota

The phylogenetic tree constructed based on the MAGs has shown that the relative evolutionary distances at the phylum level for both archaea and bacteria across various ecosystems, illustrating that several archaeal phyla, such as Halobacteriota, Thermoplasmatota, Nanoarchaeota, and Thermoproteota, were widespread in different ecosystems (Fig. S11). Notably, Halobacteriota, Nanoarchaeota, and Thermoproteota were reported to survive in extreme environments such as hot springs and saline lands [50–52]. Although limited studies suggested the presence of Thermoplasmatota on the Tibetan Plateau, our findings indicated an abundance of archaeal resources in this extreme environment, providing a novel perspective. More intriguingly, we also identified Asgardarchaeota in sediments from the Tibetan Plateau (Fig. S11).

Our analysis revealed the unique distribution pattern of Asgardarchaeota on the Tibetan Plateau. Asgardarchaeota, a phylum of Archaea, is predominantly known to inhabit hydrothermal sediments, microbial mats, as well as a range of marine environments [53]. We recovered Asgardarchaeota’s MAGs belonging to different classes from Qilian Saline Lake and Tibetan Freshwater sediments of the Tibetan Plateau, containing 9 high-quality Asgardarchaeota’s MAGs (Table S12). These findings were particularly noteworthy as they suggested that the Tibetan Plateau served as a reservoir for Asgardarchaeota (Fig. S11), highlighting its unique and pivotal role in the global distribution and evolution of this superphylum.

**Fig. 5.**
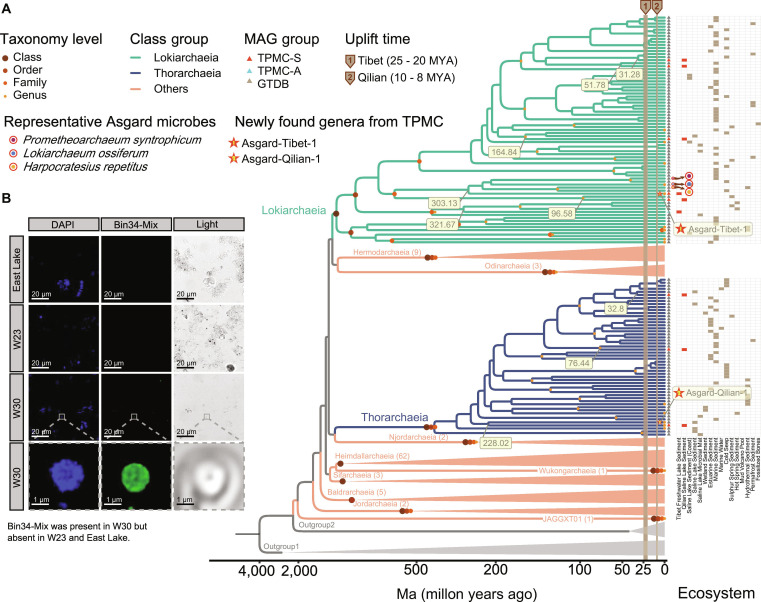
Divergence timeline of Asgardarchaeota. (A) The phylogenetic tree depicts the time-calibrated phylogeny of Asgardarchaeota, inferred from a species tree of 189 genomes utilizing 3 prior temporal constraints and an autocorrelated relaxed clock model. The numbers in the box represent the divergence time from class to genus, which is marked on the phylogenetic tree. Three classes including Lokiarchaeia, Thorarchaeia, and Heimdallarchaeia are represented by different colors. Asgardarchaeota identified through TPMC-S are highlighted with red and blue triangles. Pentagrams represent 2 newly identified genera of Asgardarchaeota from TPMC-S. Ages are represented in million year ago (Ma), tracing back from the present. The heatmap illustrates the source distribution of various Asgardarchaeota MAGs. Strips show the uplift time of the Tibetan Plateau (25 to 20 Ma) and Qilian Mountain (10 to 8 Ma), respectively. (B) Fluorescence in situ hybridization (FISH) experiment verification of Asgard-Qilian-1. Blue, DAPI (4′,6-diamidino-2-phenylindole); green, Asgard-Qilian-1 probe set (Bin34-Mix). W30, one of the most Asgardarchaeota abundant sediment sample in Qilian; W23, Asgardarchaeota lacking sediment sample in Qilian; East Lake, freshwater sample in East Lake (non-Tibetan Plateau sample). Note that a major limitation of the experiment is the absence of a cultured representative of Asgard-Qilian-1, which precluded the inclusion of a definitive positive control.

We further conducted a divergence time analysis [54] based on 207 representative Asgardarchaeota genomes from GTDB public repositories (see details in Methods; Table S12), in conjunction with representative 9 Asgardarchaeota MAGs recovered in this work (Table S12), to explore the evolution trajectories of Asgardarchaeota on the Tibetan Plateau (Fig. 5A). The majority of the MAGs belonged to classes including Heimdallarchaeia, Lokiarchaeia, and Thorarchaeia, while the Asgardarchaeota annotated in the Tibetan Plateau sediments were restricted to Lokiarchaeia and Thorarchaeia. The presence of Asgardarchaeota in sediments from the Tibetan Plateau was confirmed by fluorescence in situ hybridization (FISH) using 2 probes: a custom-designed probe named Bin80, which specifically targets one of the most abundant MAGs, and a previously reported probe specific for Lokiarchaeia [55] (see details in Methods; Table S13). Two sediments with close sampling locations (Qinghai Lake, Table S1) were selected, among which sample W30 was rich in Asgardarchaeota MAGs and sample W23 did not contain Asgardarchaeota MAGs, together with a sample from East Lake being selected as the negative control. Both probes successfully detected Lokiarchaeia in the Bin80-enriched sediment sample (W30), but not in the sample lacking Asgardarchaeota (W23) and the East Lake sample (Fig. S12). By incorporating 207 Asgardarchaeota genomes from GTDB (Table S12), along with their background information, we found that the closest evolutionary relatives of the Asgardarchaeota found on the Tibetan Plateau originated from diverse environments, including Marine sediments (3/9) and Saline Lake sediments (coast) (3/9) (Table S12). This indicated that the Asgardarchaeota in Tibetan Plateau sediments might have previously inhabited the ocean and were preserved in the sediments during the uplift of the Tibetan Plateau. This finding emphasized that the Tibet Plateau’s sediment was a reservoir abundant in Asgardarchaeota, deepening our understanding of the global distribution patterns of Asgardarchaeota.

Moreover, the most recent divergence times of Asgardarchaeota genera on the Tibetan Plateau across different ecosystems preceded the uplift of the plateau. The rapid uplift of Qilian Mountain occurred approximately 10 to 8 Ma [56], and the southern Tibetan lowland uplifted to form an exclusively high plateau more similar to that of today around 25 to 20 Ma [57]. However, most of the divergence events of Asgardarchaeota predated the uplift times of their respective habitats (Fig. 5A). This suggested that these Asgardarchaeota in the sediment had no diversification after the significant geological changes that shaped the Tibetan Plateau, indicating that they might be “refugees” that only managed to survive up to date.

Notably, 2 new genera of Asgardarchaeota, named Agard-Tibet-1 and Asgard-Qilian-1, were identified from Tibet Freshwater Lake sediment and Qilian Saline Lake sediment, respectively (Fig. 5A). We designed a probe set named as Bin34-mix consisting of 10 probes targeting sequences specifically found in Asgard-Qilian-1 (see details in Methods; Table S13). Using this probe set, we successfully detected spherical microbial cells that were exclusively present in the Asgardarchaeota-enriched sediment sample (W30), but not detected in the sediment sample lacking Asgardarchaeota (W23) and in non-Tibetan Plateau sediment sample (e.g., East Lake sample) (Fig. 5B). These findings provide evidence supporting the existence of this novel genus. The phylogenetic analysis revealed that Agard-Tibet-1 was closer to *Harpocratesius* and belonged to Lokiarchaeia, while Asgard-Qilian-1 was closer to *TEKIR-12S* belonging to Thorarchaeia, again confirming that these 2 genera might originate from the oceans (Fig. 5A). Importantly, these 2 genera were not detected in any other sources including the ocean water and ocean sediment ecosystems. Summing up, these results suggested that these 2 extinct oceanic genera might still have lived in the sediments of the Tibetan Plateau, providing avenues for the discovery of PAS outside their original habitats.

### Asgardarchaeota on the Tibetan Plateau developed unique genetic adaptations to extreme environments

**Fig. 6.**
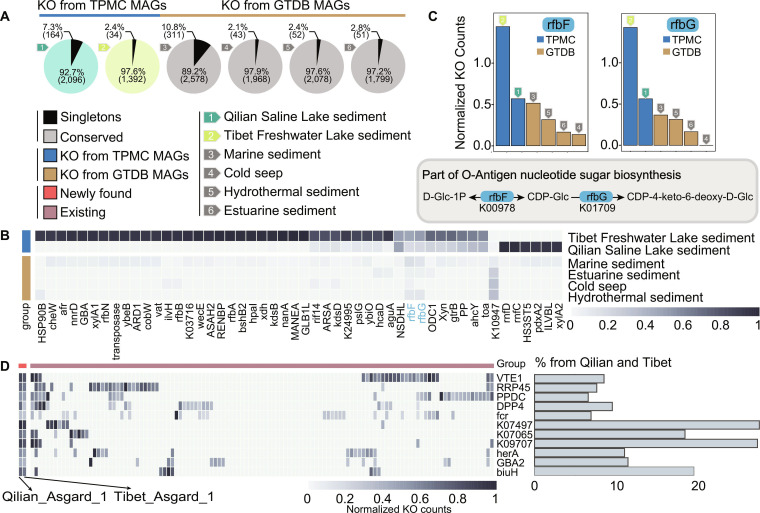
The genetic adaption of Asgardarchaeota on the Tibetan Plateau. (A) The pie charts show the number of KO on Asgardarchaeota genomes from specific ecosystems. The black portion represents the number of singletons, and the gray proportion represents conserved KO. The singletons refer to the KO only identified in one ecosystem, and the conserved KOs are those identified in at least 2 habitats. The bar charts represent the singletons and conserved KO across all 6 ecosystems. (B) Heatmap indicated the differential KO of Asgardarchaeota on the Tibetan Plateau. The *x* axis shows the symbols of differential KO, and the *y* axis shows different ecosystems. The colors of the cells in the heatmap represent the normalized KO counts (see Methods). The KOs marked in red were K00978 (rfbF) and K01709 (rfbG), which regulated the biosynthesis pathway from D-Glc-1P to CDP-4-keto-6-deoxy-D-Glc. (C) Biosynthesis pathway from D-Glc-1P to CDP-4-keto-6-deoxy-D-Glc in Asgardarchaeota on the Tibetan Plateau. Bar plots represent the normalized KO counts of enriched KO including K00978 (rfbF) and K01709 (rfbG). (D) Heatmap indicated differential KO in newly identified Asgardarchaeota genera. Each column of the heatmap represented a MAG, and each row represented a differential KO. The bar plot on the right represents the percentage of the normalized counts of this KO in the newly identified genera to the sum of the normalized counts of this KO in all Asgardarchaeotas on the Tibetan Plateau.

We next explored the genetic divergence of all the Asgardarchaeota MAGs and found that Asgardarchaeota on the Tibetan Plateau have evolved functional adaptations in response to environmental changes. Despite the fact that subregional uplift did not lead to the divergence of Asgardarchaeota at the genus level, it could be speculated that Asgardarchaeota has still developed unique genetic adaptations to cope with environmental changes. We then divided the Asgardarchaeota MAGs into 16 classes according to their respective sedimentary habitats, including Qilian Saline Lake, Tibet Freshwater, Marine, Hydrothermal, Cold seep, and Saline Lake. A total of 3,702 KEGG Orthology (KOs) were predicted from these MAGs, including 831 singletons and 2,871 conserved KO (Fig. 6A). We then conducted a differential KO analysis of Asgardarchaeota on the Tibetan Plateau to explore its unique functional evolution (see details in Methods). Most differential KOs of Asgardarchaeota on the Tibetan Plateau were enriched, with the exception of K10947, which was reduced (Fig. 6B). Notably, K00978 and K01709 were involved in the biosynthesis pathway converting D-Glc-1P to CDP-4-keto-6-deoxy-D-Glc (Fig. 6C), which were enriched in Asgardarchaeota on the Tibetan Plateau. CDP-4-keto-6-deoxy-D-Glc participates in the sugar nucleotide biosynthesis pathway [58], and the biosynthesis of nucleotide sugars in archaea facilitates the glycosylation process that contributes to the growth of halophilic archaea in the hypersaline environment [59,60].

Besides, several KOs, including K09834, K26178, K17108, K06915, and K23359, were enriched in the 2 newly found genera Asgard-Qilian-1 and Agard-Tibet-1, when compared to all the known Asgardarchaeota MAGs (Fig. 6D). Among these enriched KOs, K06915 regulates the DNA double-strand break repair helicase HerA and related adenosine triphosphatase (ATPase). HerA genes are widely conserved across archaea, and their absence severely affects UV resistance and impairs growth [61]. Intriguingly, almost all of our sampling sites were shallow sediment, and previous works have indicated that UV radiation could shape the microbial community in sediments [62,63]. It is plausible that these 2 new genera have adapted to the high UV radiation environment of the Tibetan Plateau by enhancing their DNA damage repair ability. The enrichment of the CDP-4-keto-6-deoxy-D-Glc biosynthesis pathway in the Asgardarchaeota on the Tibetan Plateau, coupled with the unique enrichment of specific KOs in the newly discovered Asgardarchaeota genera, suggested their adaptive mechanisms for osmoregulation and cryoprotection, to adapt to the high radiation and cold habitat of the Tibetan Plateau.

## Discussion

The microbial communities present in extreme environments, exemplified by the Tibetan Plateau, are under threat by human activities and global environmental changes, necessitating a full complement and urgent protection of these unique microbial resources, especially those ancient and as yet unidentified microbes. While previous efforts in soil and aquatic habitats have advanced [5,9,20,21,23], the scarcity of research on the spatial-temporal patterns of the microbial communities in aquatic and sedimentary ecosystems of the Tibetan Plateau hinders a comprehensive understanding of its microbial ecosystems. To bridge this gap, we have compiled the extensive TPMC-S based on 248 metagenomic samples of sediment in the Tibetan Plateau and systematically discovered divergence in microbial distribution patterns between sediment and aquatic ecosystems. These resources help bridge a critical knowledge gap, providing a foundation for the evidence-based prioritization of microbial conservation targets in this climate-sensitive region.

The TPMC-S served as a testament to the extraordinary microbial diversity and genomic potential within Tibetan Plateau sediment. The recovery of 5,374 novel MAGs, substantially expanding upon previous references [23,40–42], underscores sediments as a crucial frontier for microbial discovery. This aligns with findings from other extreme aquatic systems, such as high-altitude saline lakes, which are recognized as important reservoirs of novel bacterial and archaeal lineages [64,65]. Furthermore, it has uncovered 511,056,752 nonredundant genes and 26,360 predicted BGCs, the majority of which were novel, indicating that sediment was a reservoir abundant in innovative genetic and bioactive compounds. Interestingly, Saline Lake and Freshwater Lake samples exhibited the highest abundance of BGCs and enriched genes related to carbon and membrane metabolism. This pattern may reflect a dual adaptive strategy in these comparatively energy-rich but stressful habitats: (a) efficient utilization of available carbon and maintenance of membrane integrity under osmotic and thermal stress, supported by core metabolic genes, and (b) production of specialized secondary metabolites for cell protection and niche competition, facilitated by the diverse BGCs. Besides, the high abundance of BGCs, particularly Terpenes and RiPPs, points to the fact that the microbes in the sediment ecosystems have made extensive functional investments in stress protection and signaling [66–68]. Collectively, the construction of TPMC-S suggested that the Tibetan Plateau’s sediments are not merely a subset of aquatic communities but a distinct and underexplored biome harboring unique genetic resources.

Another important finding was that the similarities of sediment microbial communities followed the DDR. Geography and altitude significantly negatively affected microbial similarity, adhering to the distance–decay patterns, and emphasizing spatial factors’ role in shaping sediment microbial communities. These findings are consistent with previous studies on the soil microbiome of the Tibetan Plateau [69,70]. This pattern typically emerges from the interplay of dispersal limitation and environmental selection. The complex topography of the Tibetan Plateau likely imposes strong physical barriers to microbial dispersal, promoting divergence [71]. Concurrently, the significant environmental heterogeneity across sampled ecosystems (e.g., saline versus freshwater lakes and wetlands) acts as a powerful selective filter [72,73]. This is supported by regional studies on the Tibetan Plateau permafrost, which show that microbial specialist assemblages are particularly sensitive to local environmental gradients like pH and nutrient availability [74]. While our current data robustly establish the distance–decay pattern, fully disentangling the relative contributions of these processes from historical contingency (e.g., legacy effects of past geological or climatic events unique to each region) and priority effects (where the initial colonizing species alter the environment for later arrivals) requires future research incorporating paleoenvironmental data and temporal sampling. Nevertheless, the DDR patterns for both taxonomic and functional genes underscore that these assembly processes shape the spatial architecture of ecosystem functions, and offer valuable clues for understanding the distribution, evolution, and function of microbes in different environments on the Tibetan Plateau.

Furthermore, sediments contained a high proportion of evolutionarily PAS compared with paired aquatic samples, especially ancient archaeal lineages, than aquatic ecosystems. This supports the hypothesis that sediments function as a “sedimentary archive”. The relative stability, low energy input, and anoxic conditions of sediments may provide a refugium that favors the long-term persistence of slow-growing, oligotrophic lineages with deep evolutionary roots, in contrast to the more dynamic and competitive aquatic environment [75]. For instance, research on the cryosphere environments revealed the ancient environmental microbiomes and ancient environmental antibiotic resistance genes, and freshwater and marine sediments archive DNA from aquatic benthic and pelagic (micro)organisms [76,77]. The divergence time analysis also provides evidence to demonstrate that sediments have preferentially preserved microbial lineages whose diversification predates the dramatic uplift of the Plateau itself. This positions Tibetan Plateau sediments not only as an ecological habitat but also as a unique repository for studying deep evolutionary timelines and microbial survival across geological epochs.

Most importantly, this study has revealed that the Tibetan Plateau was a reservoir of Asgardarchaeota, and provided valuable insights into the evolutionary patterns and adaptive models of Asgardarchaeota in response to geological events. Previously characterized predominantly from marine environments [53], their presence in the Tibetan Plateau expands the known habitat range of this archaeal superphylum and suggests a remarkable biogeographic dispersal or survival history. The divergence time analysis indicating an origin predating the Tibetan Plateau uplift supports the intriguing “refugees” hypothesis—that these lineages are descendants of ancient Tethys Ocean inhabitants [78], preserved in the sediments as the plateau rose. Despite this long-term environmental stasis at the genus level, these Asgardarchaeota have still evolved unique genetic adaptations in response to environmental changes, such as the biosynthesis pathway from D-Glc-1P to CDP-4-keto-6-deoxy-D-Glc, which might be associated with the glycosylation process and facilitated their adaptation to extreme environmental conditions for osmoregulation and cryoprotection [58]. This strategy of “phenotypic stagnation and gene innovation” may be a key adaptation for long-term survival in extreme but stable environments. Microbes in other extreme environments, like Qinghai–Xizang Plateau salty lakes, are known to employ an active “salt-extraction” strategy and simultaneously accumulating compatible solutes such as glycine betaine to cope with stressors [79]. To summarize, the adaptive resilience of Asgardarchaeota to geological events underscores the imperative for conserving microbial biodiversity, as these ancient lineages hold the key to unraveling mechanisms of survival and adaptation in response to Earth’s dynamic geological history.

There are still limitations in this work. The sampling sites were concentrated in a small area of the central and northeastern Tibetan Plateau, and most samples were collected from surface sediments. More extensive and deeper sampling sites should be included in further analysis so that the samples could better represent the entire Tibetan Plateau and explore deeper microbes in the sediment. While our TPMC-S database represents a profound contribution to the field, the novelty of many of the species and genes identified underscores the need for continuous sampling and analysis. Besides the effect of geographical and vertical distances, environmental factors (e.g., pH, temperature, and salinity) could also shape the structure and function of microbial communities in different regions, which need further exploration. Only CheckM was used for the genome binning assessment, novel species were probably underestimated in completeness or overestimated in contamination rates due to the insufficient coverage of marker genes in its database, and more validating tools should be used to reduce errors. Although the identification of Asgardarchaeota MAGs and FISH experiment verification supported the existence of Asgardarchaeota in the Tibetan Plateau, future isolation or additional genomic and cytological comparisons were still needed for more robust validation. Moreover, although the identification of Asgardarchaeota on the Tibetan Plateau is interesting, the current sampling depth is limited to the surface of the Tibetan Plateau. Whether Asgardarchaeota exists in deeper sediments still needs further exploration.

In conclusion, our study has compiled a comprehensive microbial catalog of sediments in the Tibetan Plateau, uncovered extensive microbial and genetic diversity, and demonstrated the distance–decay distribution pattern for these microbial communities within the sediments. Importantly, we have found that sediments preserve more PAS surving as a “sedimentary archive”, and identified the sediments as a reservoir for Asgardarchaeota. Although their differentiation predated the subregional uplift of the Tibetan Plateau, they have adapted genetically to environmental changes. Our findings enhanced the understanding of the microbial patterns in the extreme environment and their links to changes in the natural geographic environment, and emphasized the importance of evolutionary legacy preservation in microbial conservation.

## Methods

### Sample collection

A total of 248 metagenomic samples from sediments of the central plateau of the Tibetan Plateau (abbreviated as Tibet, *n* = 175) and the northeastern margin of the Tibetan Plateau (Qilian Mountains–Qinghai Lake, abbreviated as Qilian, *n* = 73) were collected in this study. These samples included different ecosystems, including Saline Lake (*n* = 59), Freshwater Lake (*n* = 42), River (*n* = 46), and Wetland (*n* = 101). The sample sources of Qilian (sediment) were River (*n* = 23), Saline Lake (*n* = 38), and Wetland (*n* = 12), and the sample sources of Tibet (sediment) were Freshwater Lake (*n* = 42), River (*n* = 23), Saline Lake (*n* = 21), and Wetland (*n* = 89).

We also paired 241 aquatic samples on the Tibetan Plateau from our previous study [23] with the sediment samples for comparison, which were sourced from identical collection sites but varying in depth (Tables S1 and S2). These samples included different ecosystems, including Saline Lake (*n* = 66), Freshwater Lake (*n* = 49), River (*n* = 30), and Wetland (*n* = 96). The sample sources of Qilian were River (*n* = 16), Saline Lake (*n* = 36), and Wetland (*n* = 10), and the sample sources of Tibet were Freshwater Lake (*n* = 49), River (*n* = 14), Saline Lake (*n* = 30), and Wetland (*n* = 86).

### DNA extraction and metagenome sequencing

The cetyltrimethylammonium bromide (CTAB) method was used to extract environmental DNA from the filter membrane. First, the membrane was cut into small pieces using scissors (cut more than 8 times). Subsequently, the cut membrane was placed in a 1.5-ml Eppendorf (EP) tube, and 800 μl of CTAB extraction buffer [2% CTAB, 100 mM tris–HCl (pH 8.0), 20 mM EDTA, 1.4 M NaCl] was added for mixing. Then, grinding beads were added to it, and after grinding using a grinder (Tissuelyser-192L, Jingxin, Shanghai, China, 55 Hz, 5 min), it was heated at 70 °C for 10 min in a water bath to ensure sufficient DNA extraction. Following the extraction of DNA using separation methods, an equal volume of chloroform:isoamylalcohol (24:1) was used to remove proteins. The DNA degradation degree and potential contamination were monitored on 1% agarose gels. The DNA purity was determined using the NanoPhotometer spectrophotometer (IMPLEN, CA, USA), and the DNA concentration was measured using the Qubit dsDNA Assay Kit in Qubit 2.0 Fluorometer (Life Technologies, CA, USA). One microgram of qualified DNA was used to construct the library. For high-quality DNA, the Covaris (Covaris Inc., Woburn, MA, USA) instrument was used to fragment DNA into approximately 350-base pair (bp) fragments, followed by end repair and addition of “A” tails to the 3′ end. The Illumina sequencing adapters were then ligated to both ends of the library DNA using T4 ligase. After library quality control, the libraries were sequenced on the Illumina MGISEQ 2000 Platform (China National GeneBank, Shenzhen, China). Sequencing adapters of the paired-end reads were removed using the SOAPnuke filter (version 2.1.5).

### Contig assembly and ORF prediction

The contig assembly and gene ORF prediction were consistent with the previous study of TPMC-A [23]. Metagenome reads were assembled into contigs using Megahit [80] (version 1.1.3), with default parameters. Contigs with length ≥1,000 bp were retained. Eukaryotic contigs were identified by cutting the contig into sub-contigs (windows = 1 kb, steps = 0.5 kb) and searching them against the NR (ftp://ftp.ncbi.nlm.nih.gov/blast/db) using DIAMOND (version 2.1.6) [81] BLASTX module. If 60% of the sub-contigs within one contig had the best hit as eukaryotic origin, the entire contig was recognized as eukaryotic origin, as previously described [2]. Gene ORFs for the metagenomic assemblies were predicted using Prodigal (version 2.6.3) [82] with parameters “-p meta”. Gene ORFs with length <100 bp were removed.

### Nonredundant gene catalog construction

A total of 701,528,671 gene ORFs were de-replicated by clustering at 80% aligned region with 95% nucleotide identity using MMseqs2 (version 13.45111) [83], with the parameters “easy-linclust -e 0.001, --min-seq-id 0.95”, as used in the datasets of the TPMC-A, TG2G, and human gut microbiome datasets [2,23,84], and “-c 0.80” was set to exclude the effects of the shorter genes [2]. Finally, a total of 296,289,678 nonredundant genes were obtained. The gene rarefaction analysis was performed by sampling the whole gene set to count the number of the gene clusters produced by MMseqs2, with a 5% sampling step 10 times.

### Gene catalog abundance and annotation

The abundance profile of the nonredundant gene catalog was produced using Salmon (version 1.10.1)[85]: (a) An index was built for the gene catalog using “salmon index” with default settings, and (b) quality-controlled metagenomic reads for each sample were quantified using “salmon quant” with the “-validateMappings” flag. For cross-sample comparisons, the abundance profiles were normalized by the library size. The nonredundant gene catalog was functionally annotated against the NR and Swiss-Prot [43] databases using MMseqs2, with parameters “easy-search -e 0.01, --min-seq-id 0.3, --cov-mode 2, -c 0.8”, as used in the TPMC-A and TG2G datasets [2,23]. In addition, the de-replicated genes were also functionally annotated against the eggNOG Orthologous Groups database [86] (version 5.0) using eggNOG-mapper [87] (version 2.0.1), which integrates functional annotations from several sources, including the KEGG functional orthologs database [45], the carbohydrate-active enzymes (CAZy) database [46], and Cluster of Orthologous Groups categories (COG) database [44]. Moreover, antibiotic resistance genes were annotated by querying the de-replicated protein sequences against the Comprehensive Antibiotic Resistance Database (CARD, version 3.2.5) [47] using Resistance Gene Identifier (RGI, version 6.0.1) [88], with parameters “-t protein, -a DIAMOND, --include_loose, -d wgs”. Furthermore, virulence factors were annotated by aligning gene sequences against the VFDB 2023 [48] using DIAMOND blastp (version 2.1.6) [81] with an *e*-value threshold of 1 × 10^−5^. The genes that failed to be mapped to any of these databases were designated as novel genes.

### MAG construction and genome quality control

MAGs were built and refined using MetaWRAP (version 1.3.0) [89] with default parameters, which integrated the binning results of metaBAT2 (version 2.12.1) [90], MaxBin2 (version 2.2.6) [91], and CONCOCT (version 1.0.0) [92]. The completeness, contamination, and strain heterogeneity were calculated using the “lineage_wf” module of CheckM (version 1.2.2) [93] with default parameters.

A thorough taxonomy-based post-binning refinement process was then implemented for each MAG meeting the criteria of completeness ≥ 50% and contamination < 10%. This process involved 2 key steps: (a) Removal of contigs identified as eukaryotic origin or viral origin. Eukaryotic contigs were identified as described above. Viral contigs were identified by VirSorter2 (version 2.2.4) [94], with parameters “--include-groups dsDNAphage, NCLDV, RNA, ssDNA, lavidaviridae, --min-length 5000, and--min-score 0.5”. (b) Removal of contigs originating from a different species compared to the dominant organism present in the MAG, using MAGpurify (version 2.1.2) [95] with default modules and parameters (“phylo-markers”, “clade-markers”, “tetra-freq”, “gc-content”, and “known-contam”). This refinement process has removed a total of 73,230 contigs, constituting 1.02% of the initial 7,183,800 contigs within the bins.

CheckM (version 1.2.2) [93] was then re-conducted on these refined mags to calculate the completeness, contamination, and strain heterogeneity. The presence of rRNA and tRNA genes was identified using Infernal (version 1.1.3) [96] with models from the Rfam database [97] and the parameters “--cut_ga, --rfam”. According to the standard of the MIMAG [98], only the refined MAGs meeting the medium and higher quality remained for the subsequent analysis, which was defined as follows: (a) medium-quality MAGs: completeness ≥ 50% and contamination < 10%, and (b) high-quality MAGs: completeness > 90% and contamination < 5% with the presence of the 23S, 16S, and 5S rRNA genes and at least 18 tRNAs.

### MAG clustering and taxonomy annotation

The 13,696 refined MAGs were clustered into 6,233 representative genome-resolved species using dRep (version 3.4.2) [99] based on >30% AF and a genome-wide ANI threshold of 95%, with parameters “-nc 0.3 and -sa 0.95”, as used in the genomic catalog of TPMC-A [23], TG2G [2], GEM [41], and human gut microbiomes [84]. The taxonomy annotation of the 6,233 species was performed using the module “classify_wf” of Genome Taxonomy Database Toolkit (GTDB-Tk, version 2.2.6) [100] against the GTDB release R214 [40] with default parameters. The phylogenetic tree was generated through the maximum-likelihood placement of each MAG of this study in the GTDB-Tk reference tree using pplacer [101]. The subsequent visualization was achieved through ITOL (version 6) [102].

### Microbial community diversity analysis

The taxonomic abundance profile of TPMC-S microbial communities (*n* = 248) for diversity analysis was quantified using the CoverM pipeline (v0.7.0) as previously described [103]. Metagenome reads were mapped against 6,233 representative genome-resolved species, applying stringent alignment criteria with parameters “--min-read-percent-identity 95” and “--min-read-aligned-percent 75”. To ensure consistency in cross-sample comparisons and to measure the relative abundance of MAGs, the effects of sequencing depth and MAG size on abundance were corrected by “-m TPM”.

Shannon index was employed to assess microbial community diversity across ecosystems. Beta diversity was displayed through principal coordinate analysis (PCoA) based on the Bray–Curtis dissimilarity metric. To further explore the ecological processes, the relationship between occurrence frequency and relative abundance of genome-resolved species, recognized as NCM, was modeled using nonlinear least squares fitting across all samples. This approach allowed for the estimation of genome-resolved species migration rates. The coefficient of determination (*R*^2^) was utilized to evaluate the goodness-of-fit of NCM.

### Novelty of MAGs

The representative MAGs of the genome-resolved species were compared against 85,205 representative genomes from GTDB release R214 [40], 22,746 representative genomes of environmental origin from GEM [41], 21,077 representative genomes from SMAG [42], and 10,723 representative genomes from TPMC-A [104] using the “compare” module of dRep. The MAGs exhibiting an ANI < 0.95 and a coverage < 0.3 compared to the reference genomes were designated as novel.

### Identification and clustering of BGCs

To retain a complete biosynthetic resource of the TPMC-S, all the initial medium-quality MAGs (*n* = 14,196) were used, with the removal of the contigs from eukaryotic or viral origins. A total of 26,360 BGCs were predicted and identified on contigs ≥ 5 kb of the MAGs to reduce the risk of fragmentation [16], using AntiSMASH (version 6.1.1) [105], with parameters “--minlength 5000, --genefinding-tool prodigal-m”. Each BGC was functionally annotated based on the predicted product types as defined by AntiSMASH and BiG-SCAPE, and categorized into 8 groups using BiG-SCAPE (version 1.1.5) [106]: “PKSI”, “PKSother”, “NRPS”, “RiPPs”, “Saccharides”, “Terpene”, “PKS-NRP_Hybrids”, and “Others”. BGCs were then clustered into 10,888 GCFs (0.3 distance threshold) and 453 GCCs (0.7 distance threshold) using BiG-SCAPE [106] with default parameters. To prevent sampling biases in quantitative analysis (taxonomic and functional compositions of GCCs/GCFs, GCF, and GCC distances to reference databases as well as GCF metagenomic abundances), the 26,360 BGCs were further dereplicated by retaining only the longest BGC per GCF per species [16], resulting in a total of 14,109 BGCs. The abundance of each of the biosynthetic genes of a BGC was calculated using Salmon (version 1.10.1) [85] and then normalized by the library size as described in the gene-level profiling methods. The metagenomic abundance of a BGC was estimated as the median abundance of its biosynthetic genes (as defined by antiSMASH). The metagenomic abundance of each GCF and GCC was then computed as the sum of its representative BGCs.

### Novelty of BGCs, GCFs, and GCCs

The novelty of BGCs, GCFs, and GCCs was determined by querying the 14,109 representative BGCs against a pre-processed BiG-FAM [107] reference database, using BiG-SliCE (version 1.1.0) [49] with default parameters. This reference database contained 1,225,071 BGCs from 209,206 publicly available microbial genomes, which integrated MIBiG v2.0 [108], RefSeq complete/draft bacteria, GenBank fungi, Genbank archaea, and other MAGs from different studies. The reference database had generated 29,955 GCF reference models. The rank first GCF of each query BGC with the calculated membership value ≤ 900 as used in BiG-FAM was designated as the matched GCF. The query BGCs that failed to map any of the reference GCFs were designated as novel. The query GCF with the proportion of novel BGCs < 0.2 was designated as the novel, while the query GCC with that proportion < 0.4 was designated as the novel.

### Construction of phylogenetic tree for divergence time analysis

The divergence time phylogenetic tree was estimated using RelTime [109], based on the topological structure and multiple sequence alignment (MSA) constructed from the nonredundant MAGs (*n* = 11,512) from TPMC-A and TPMC-S, as derived from the GTDB. The phylogenetic trees were inferred using FastTree with the LG model, supported by the Shimodaira–Hasegawa (SH) test. Divergence time estimates were subsequently made separately for archaea (*n* = 197) and bacteria (*n* = 11,316), employing distinct markers for each domain. Roots were introduced from outside, with “Methanopyrus kandleri” as the archaea and “Gloeobacter violaceus PCC 7421” as the bacteria. We used intervals as calibration points for the divergence time range of Archaea and Bacteria, respectively (Table S14). Given the difficulty of molecular dating with data matrices containing hundreds of species and thousands of genes, the large bacterial data matrix (11,316 species × 120 genes) posed particular challenges in estimating divergence times. To address this, we merged phyla with fewer than 30 MAGs, with each phylum assumed to follow a normal distribution, approximated by the central limit theorem. A stratified sampling approach was employed to determine the distribution of 200 MAGs, drawn from different ecosystems (TPMC-S and TPMC-W), with each distribution sampled from a normal distribution, and the process was repeated 15 times. For each iteration of the phylogenetic tree construction, an amino acid substitution model (LG + F) was used, combined with a gamma distribution model and an invariant sites model (G + I) to account for rate variation across different positions in the sequences.

### Construction of the phylogenetic tree of Asgardarchaeota MAGs

We downloaded all high-quality Asgardarchaeota MAGs (completeness > 80%, contamination < 10%) from GTDB as ref_Asgardarchaeota and predicted the corresponding protein sequences using Prodigal. We then obtained ribosomal protein (RP) sequences shared by the 3 domains of life (bacteria, archaea, and eukaryotes), which were aligned using Mafft with default parameters [110]. Based on the alignment, we generated a position-specific scoring matrix (PSSM) for the 3-domain RPs using PSI-Blast [111]. PSI-Blast was run without limiting the number of iterations, and the resulting PSSM matrix was used to search for RP sequences from both TPMC_ Asgardarchaeota and ref_ Asgardarchaeota, yielding an initial pool of Asgardarchaeota RP sequences.

Next, we constructed a BLAST database containing the shared RP sequences from the 3 domains, and performed a reverse BLAST search of the initial Asgardarchaeota RP sequence pool against this database, setting the threshold to 1e−4. From the reverse BLAST results, we extracted the best-hit RP sequences for each MAG and category, which served as input for phylogenetic tree construction.

The best-hit sequences were aligned using Mafft, and the alignment was subsequently trimmed with BMGE to remove low-quality regions [112]. Sequences from the S14 class, which had fewer species representatives, were also removed. The trimmed sequences were concatenated to maximize their utilization in the subsequent analyses. PartitionFinder was used for partitioning optimization to identify the best model partitioning scheme [113]. Based on this scheme, IQ-TREE was employed to determine the most appropriate substitution models for each partition [114]. Using the optimal partitioning and substitution models, we conducted a symmetry test with the “--symtest-remove-bad” option in IQ-TREE to ensure that no unreliable branches were present in the phylogenetic tree. Finally, we performed 1,000 ultrafast bootstrap replicates in IQ-TREE to construct the phylogenetic tree and conducted SH-aLRT testing.

For divergence time estimation, 3 constraints were applied: (a) the minimum origin of oxygenic cyanobacteria was set to 3,000 Ma; (b) the minimum origin of Nostocales was set to 1,600 Ma; and (c) the maximum age of the most recent common ancestor of *Sulfolobus solfataricus* and *Sulfolobus islandicus* was set to 47.5 Ma. After midpoint rooting of the phylogenetic tree, these time constraints were input into RelTime for divergence time inference.

### Probe design and FISH experiment

To design FISH probes, we initially attempted to identify repetitive sequences unique to Bin80 (a MAG belonging to Lokiarchaeia) and Asgard-Qilian-1 (a novel genus within Thorarchaeia) using RepeatModeler (v2.0.5) [115]. The goal was to generate probes that target multiple loci within each MAG, thereby enhancing fluorescence signal intensity. For Bin80, we identified a promising sequence containing 13 repeats, which was used as the probe (Table S13). Additionally, a previously reported Lokiarchaeia-specific probe was synthesized for verification (Table S13). For Asgard-Qilian-1, no suitable repetitive sequences were identified using the above strategy. As an alternative, we designed a probe set (Bin34-Mix) consisting of 10 individual probes with length ranging from 20 to 30 nucleotides, each targeting a distinct genomic locus in Asgard-Qilian-1 (Table S13). To do this, contigs from Asgard-Qilian-1 were segmented into short sequences using a sliding window approach (window size = 30 bp, step size = 15 bp). These short sequences were then searched against the TPMC-S database using BLASTN (v2.10.1) [116] with the parameters “-task blastn-short -word_size 7”. Nonspecific sequences were filtered out based on the following criteria: *E* < 20, alignment coverage ≥ 60%, and sequence identity ≥ 90%. The remaining candidate sequences were further aligned against the National Center for Biotechnology Information (NCBI) nt database (as of 2024 December 31) to remove fragments with significant similarity to known archaeal sequences. Subsequently, BLAT (v35) [117] was used to compare the filtered sequences against both the GTDB database (r220) [100] and the TPMC-S Asgard MAG database. Only unique sequences without cross-genus matches were retained. These strategies ensured that this probe was unique to Asgard-Qilian-1, as regard to all microbial species information from the TPMC-S, GTDB, and NCBI nt databases.

The 100-μl sediment sample was transferred into a 1.5-ml centrifuge tube. Then, 1 ml of phosphate-buffered saline (PBS) was added, and the mixture was gently pipetted to homogenize the sample. The suspension was centrifuged at 500*g* for 2 min to remove large particles. The resulting supernatant was transferred to a new tube and centrifuged at 20,000*g* for 2 min to enrich microbial cells. The resulting pellet was resuspended in 2% paraformaldehyde (PFA) in 1× Pipes, Hepes, EGTA, and MgSO4 (PHEM) Buffer and fixed at 4 °C for 1 h. The fixed cells were adhered to the slide. The sample was washed twice with 1× PBS for 5 min each time. The FISH steps refer to the Direct Fluorescence Bacterial In Situ Hybridization Test Kit (FUTURE BIOTECH, catalog no. FB0016) kit. The detailed steps are as follows: add 100 μl of solution A to cover the sample, stand at room temperature for 15 min; absorb solution A, add 100 μl of solution B, stand at room temperature for 15 min; absorb solution B, add 100 μl of 1× PBS to wash the cells for 5 min; add 100 μl of solution C, stand at room temperature for 15 min; then soak in 1× PBS solution for 5 min; add 100 μl of Blocking Buffer to the specimen, place in a wet box, and incubate at a constant temperature of 55 °C for 2 h. The probe was diluted with 45% Hybridization Buffer at a ratio of 1:100, denatured at 88 °C for 3 min, and equilibrated at 37 °C for 5 min; 30 μl of probe solution was added; a coverslip was placed on top; and the coverslip was sealed with Fixo gum around it and hybridized in a wet box at 37 °C for 24 h. The coverslip was removed, and the slide was placed into 1× Washing Buffer I for a 5-min wash, then moved to a new Washing Buffer I preheated to 60 °C for a 2-min wash, and finally moved to room-temperature 1× Washing Buffer II working solution for a 15-min wash. Thirty microliters of 5 μg/ml DAPI (4′,6-diamidino-2-phenylindole) was added to cover the sample and incubated in the dark at room temperature for 15 min. The specimen was sequentially immersed in 70%, 85%, and 100% ethanol, each dehydrated for 2 min, air-dried at room temperature; 15 μl of MOUNT SOLID ANTIFADE (Abberior, catalog no. MM-2013-2315ML) was dropped, the coverslip was covered, and the edges of the coverslip were sealed with Fixo gum. Fluorescence staining was observed at 100× magnification, and images were recorded with a Leica TCS SP8 scanning confocal microscope with LIGHTNING (Leica Microsystems, Mannheim, Germany).

### Identification of differential pathways

KO of each MAG was enriched from the predicted genes. (a) Standardization of the abundance of KO: The number of KO in each MAG was divided by the size of the MAG’s size to get the abundance of KO per unit size MAG of each MAG; then the abundance of KO per unit size MAG of MAGs belonging to the same ecosystems was added and divided by the number of MAGs of this ecosystem to obtain the abundance of KO per unit size MAG of this ecosystem. (b) Prevalence of the KO: KOs that existed in more than 30% of 14 ecosystems (except for Qilian Saline Lake sediment and Tibet Freshwater Lake sediment) were defined as prevalent KOs, and prevalent KOs were retained for the next analysis. (c) Enrichment or reduction of KO: Subtracted the abundance of each KO per unit size MAG of the other 14 ecosystems from that of Qilian Saline Lake sediment, and then divided by the summary of the abundance of this KO per unit size MAG of Qilian Saline Lake sediment and 14 other ecosystems. If the ratio > 0.9, this KO will be defined as enriched KO of the Qilian Saline Lake sediment; if the ratio < −0.9, this KO will be defined as reduced KO of the Qilian Saline Lake sediment. (d) Enrichment of differential pathways: The differential pathways were obtained based on the enrichment of differential KO.

### Quantification and statistical analysis

All *P* values were adjusted by Benjamini–Hochberg methods. Significance was determined at an adjusted *P* < 0.05 between groups.

## Data Availability

The metagenomic sequencing data of TPMC-S generated in this study have been deposited in the Genome Sequence Archive (GSA) section of the National Genomics Data Center under accession code PRJCA036742. The TPMC-S database is available at https://ngdc.cncb.ac.cn/tpmc-s, including all the metadata of the 248 sediment metagenomic samples, TPMC-S nonredundant gene sequences and their annotations https://download.cncb.ac.cn/bigd/TPMC-S/TPMC_sediment_gene_catalog/, metagenome-assembled genomes and their annotations https://download.cncb.ac.cn/bigd/TPMC-S/TPMC_sediment_genome_catalog/, as well as BGCs and their annotations https://download.cncb.ac.cn/bigd/TPMC-S/TPMC_sediment_BGC/. The referenced representative genomes including 85,205 from GTDB release R214 [40], 22,746 of environmental origin from GEM [41], 21,077 from SMAG [42], and 10,723 from the TPMC-A [23], are available at https://gtdb.ecogenomic.org/, https://portal.nersc.gov/GEM/genomes/, https://microbma.github.io/project/SMAG.html, and https://download.cncb.ac.cn/bigd/TPMC/TPMC_genome_catalog/, respectively.
