# Supplementary material for: Data Mining of Sediment Microbiomes of the Tibetan Plateau Revealed a Genomic Repository of Ancient Lineages and Adaptive Evolution of Asgardarchaeota
Source: Research (Wash D C). 2026 Mar 31;9:1213. doi: 10.34133/research.1213 (PMC13036324; doi:10.34133/research.1213)
Supplement: Supplementary 1 — Figs. S1 to S12 Tables S1 to S14 [file research.1213.f1.zip › supplementary materials 20260111.docx]

Supplementary materials for

**Sediment microbiomes of the Tibetan Plateau: a genomic repository of ancient lineages and adaptive evolution of Asgardarchaeota**

# Supplementary Figures


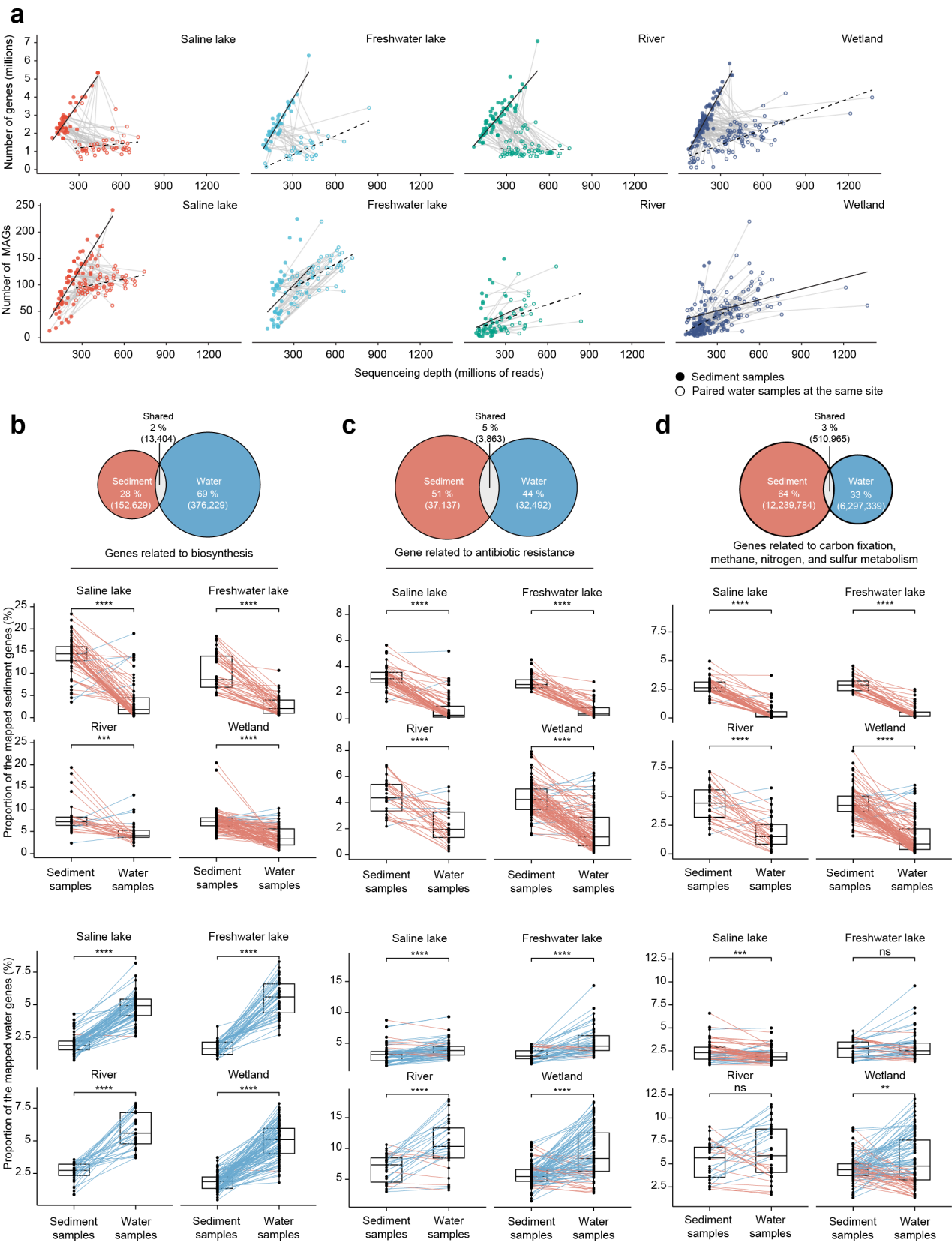


**Figure S1 | Functional disparity between sediment and aquatic samples. a** Differences in number of predicted genes and recovered MAGs between pairs of sediment-water samples against sequencing depth. The paired samples are connected by grey lines. The solid line represents the relationship between the sequencing depth and the number of genes or MAGs of sediments samples, and the dashed line represent the relationship between the sequencing depth and the number of genes or MAGs of aquatic samples. **b**–**d** Venn diagrams show the shared and specific genes related to biosynthesis (b), antibiotic resistance (c), and carbon, nitrogen, and sulfur metabolism (d) between the TPMC-S and TPMC-A catalogs. The boxplots show the proportion of the TPMC-S genes mapped to sediment samples and the paired water samples, as well as the TPMC-A genes mapped to sediment samples and the paired water samples at the same site, respectively. The statistical significance is calculated by the two-sided paired Mann-Whitney-Wilcoxon test. Boxes represent the interquartile range between the first and third quartiles and the line inside represents the median. Whiskers denote the lowest and highest values within the 1.5×interquartile range from the first and third quartiles, respectively. The paired samples are connected by lines, where blue lines represent higher abundances in sediment samples, while red lines indicate lower abundances in sediment samples.

**
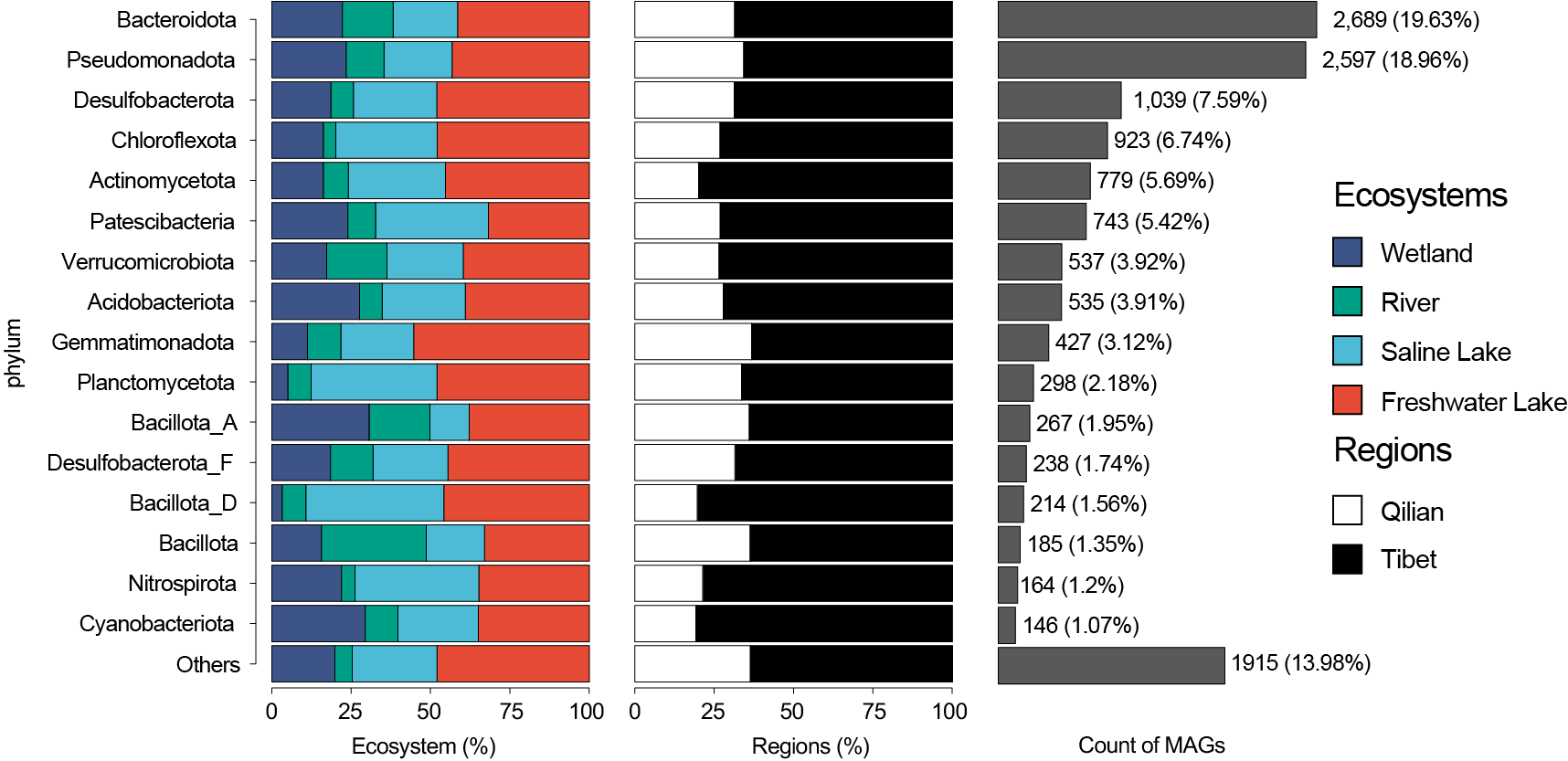
**

**Figure S2 | Environmental and geographical distribution of genome-resolved species.** The left panel lists phyla in descending order of relative abundance. Phyla with <1% number of MAGs were designated as “Others”. Central two percentage stacked bar charts quantify the relative frequency of BGC types across the phyla in ecosystems and regions. The right gray bars indicate MAG counts and their proportional representation in the total TPMC-S dataset.


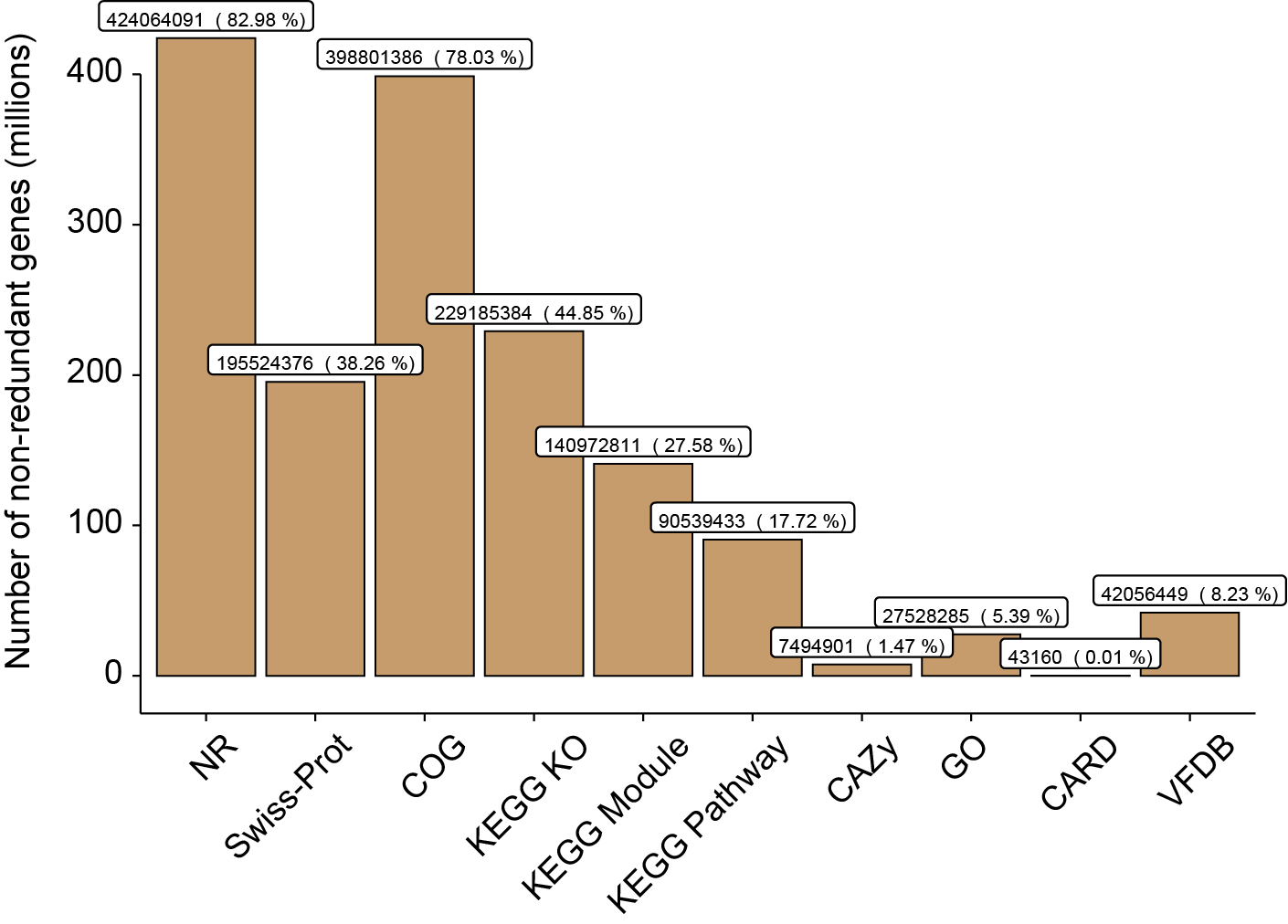


## Figure S3 | Functional annotations of the TPMC-S gene catalog. The numbers shown represent the number of non-redundant genes mapped to the public databases. X-axis represents the databases used for comparison, and Y-axis represents the number of non-redundant genes of TPMC-S compared with these databases.


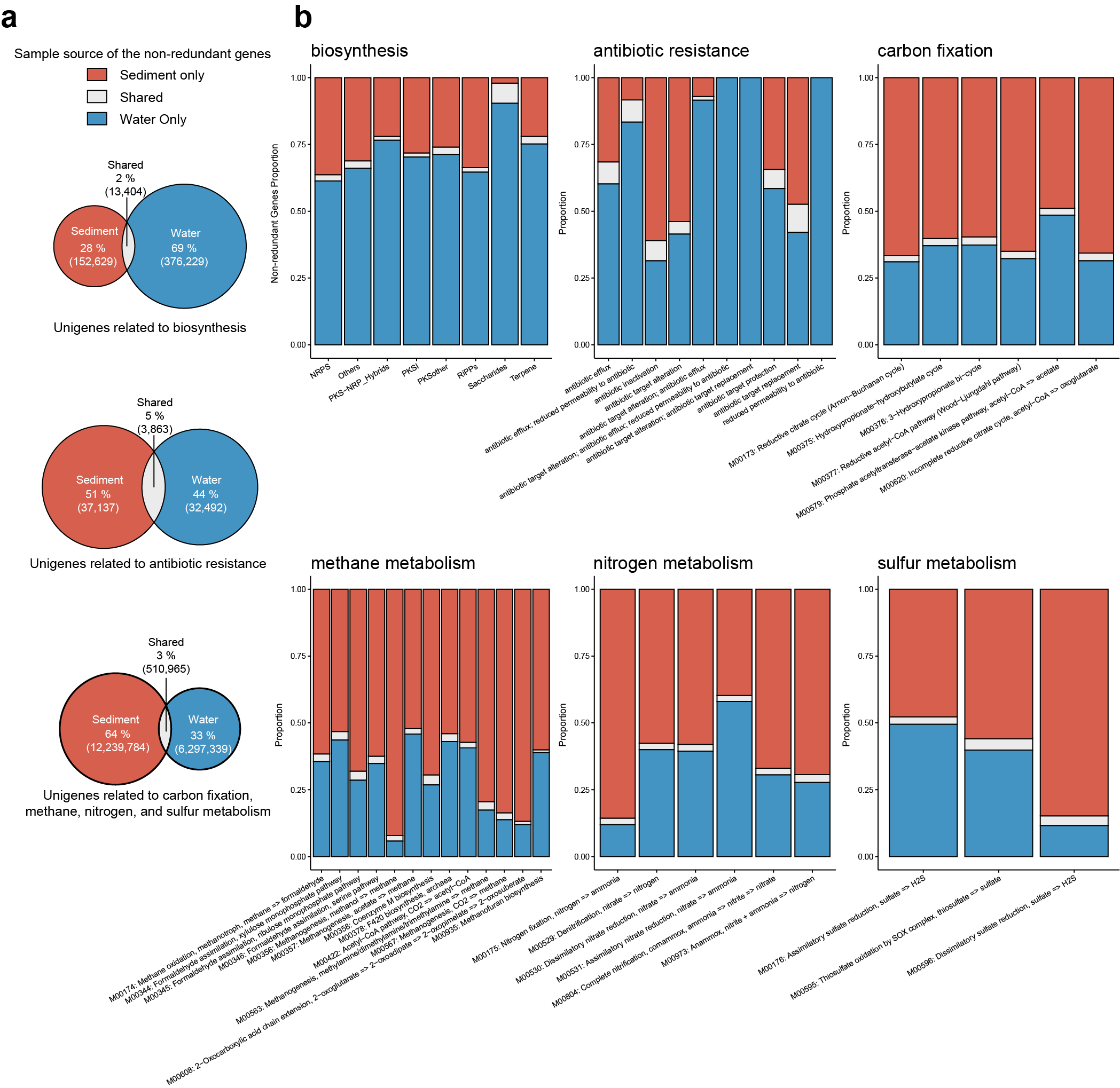


**Figure S4 | Proportion of functional genes shared between sediment and aquatic samples. a** Venn diagrams show the shared and specific genes related to biosynthesis, antibiotic resistance, and carbon, nitrogen, and sulfur metabolism between the TPMC-S and TPMC-A catalogs. **b** The stacked bar plots show the proportion of detailed types of functional genes between TPMC-S and TPMC-A.


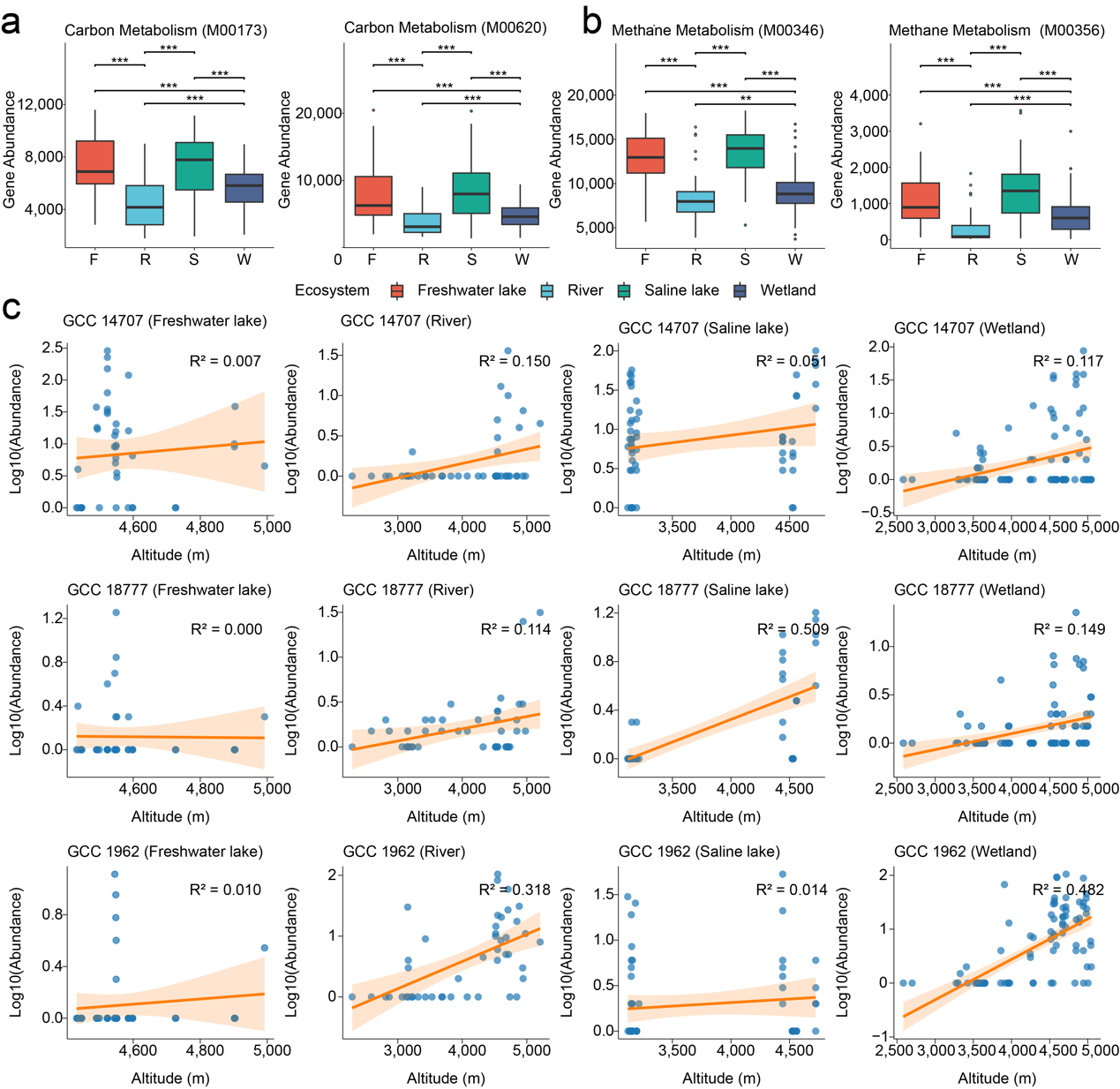


**Figure S5 | Regional variations in metabolic profiles.** **a** The abundance of representative carbon metabolic modules (M00173 and M00620) across ecosystems. **b** The abundance of representative methane metabolic modules (M00346 and M00356) across ecosystems. NS, no significance; *, *P* < 0.05; **, *P* < 0.01; ***, *P* < 0.005 ****, *P* < 0.001. **c** Linear fitting (orange line) of altitude and representative GCC abundance (GCC 14707, GCC 18777, and GCC 1962) across ecosystems.


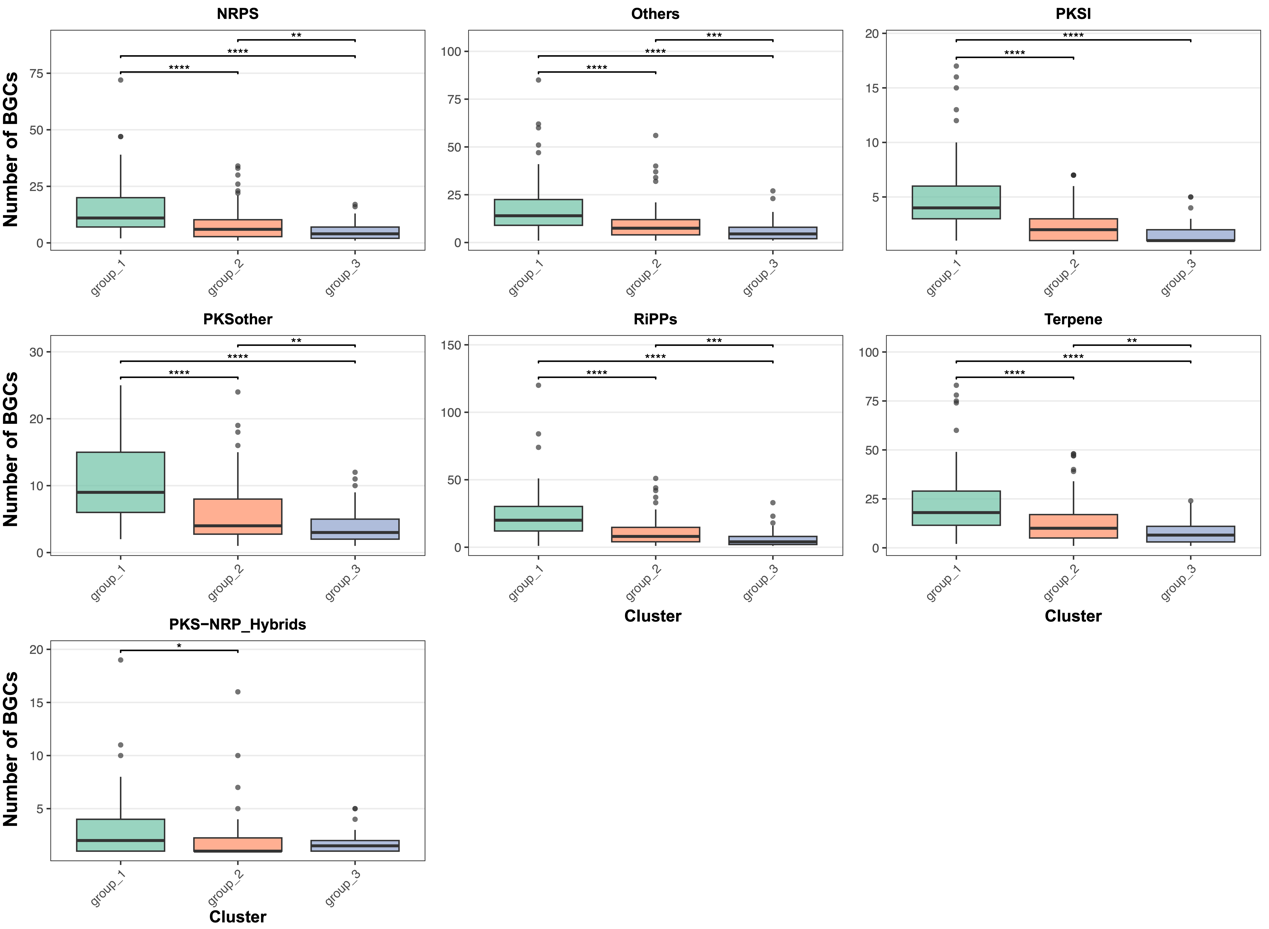


**Figure S6 | The number and class of BGCs in three clusters.** Inter-group differences were evaluated with Wilcoxon test. NS, no significance; *, *P* < 0.05; **, *P* < 0.01; ***, *P* < 0.005 ****, *P* < 0.001.

**~~
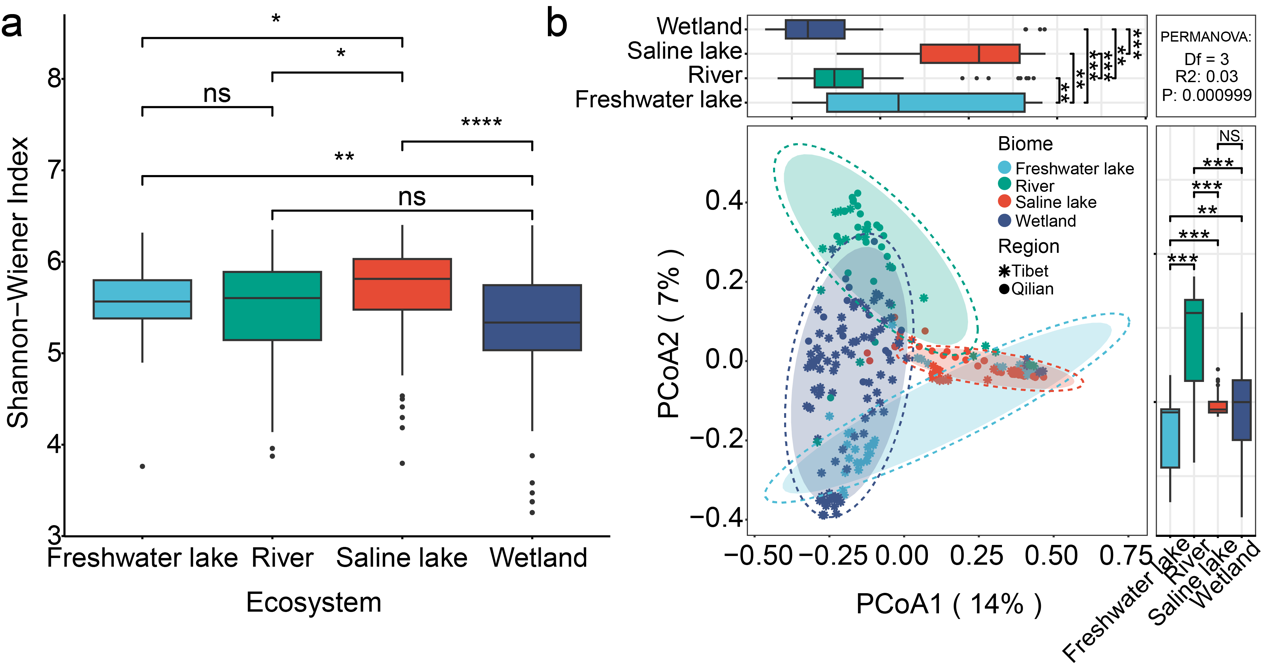
~~**

**Figure S7 | Microbial community analysis across ecosystems. a** Boxplots of Shannon-Wiener diversity index distributions for four sediment ecosystems of the Tibetan Plateau. Whiskers denote the lowest and highest values within the 1.5×interquartile range from the first and third quartiles, respectively. **b** Principal coordinate analysis (PCoA) based on Bray-Curtis distances, showing β-diversity partitioning among ecosystems. The ellipses indicate 95% confidence intervals. The colors of dots represent ecosystem types, and the shapes of dots represent regions. Overall differences are assessed using PERMANOVA, and inter-group differences were evaluated with Wilcoxon test. NS, no significance; *, *P* < 0.05; **, *P* < 0.01; ***, *P* < 0.005 ****, *P* < 0.001.


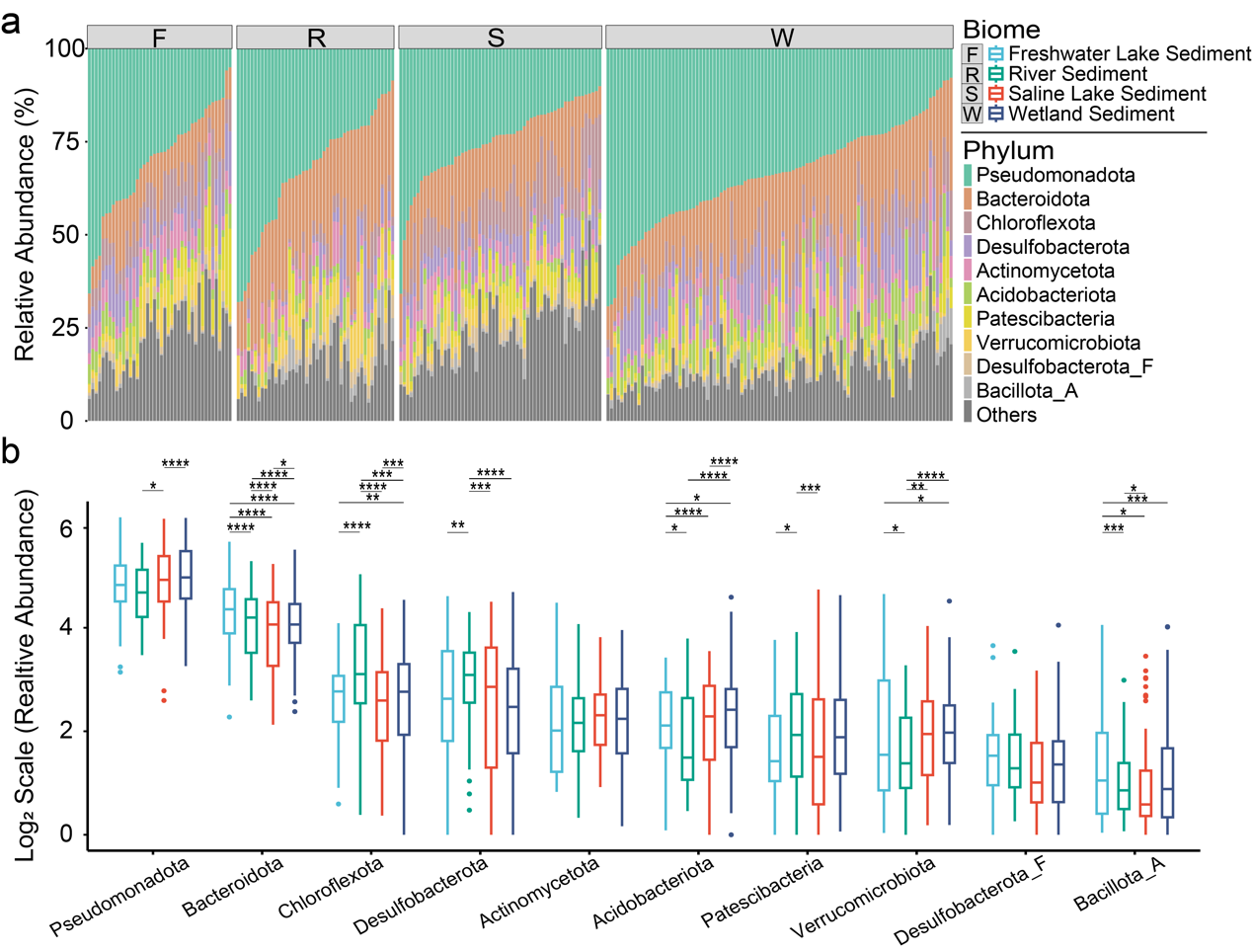


**Figure S8 | Composition and comparative analysis of dominant bacterial phyla between TPMC-S and TPMC-A. a** Stacked bar plots show the relative abundance of the top 10 bacterial phyla across four ecosystems, with remaining phyla aggregated as “Others”. The color blocks represent distinct phyla. **b** Boxplots display the distribution of the top 10 phyla, with median values shown as horizontal lines and interquartile ranges as boxes. Significant inter-ecosystem differences are calculated based on Wilcoxon test. Whiskers denote the lowest and highest values within the 1.5×interquartile range from the first and third quartiles, respectively. *, *P* < 0.05; **, *P* < 0.01; ***, *P* < 0.005 ****, *P* < 0.001.


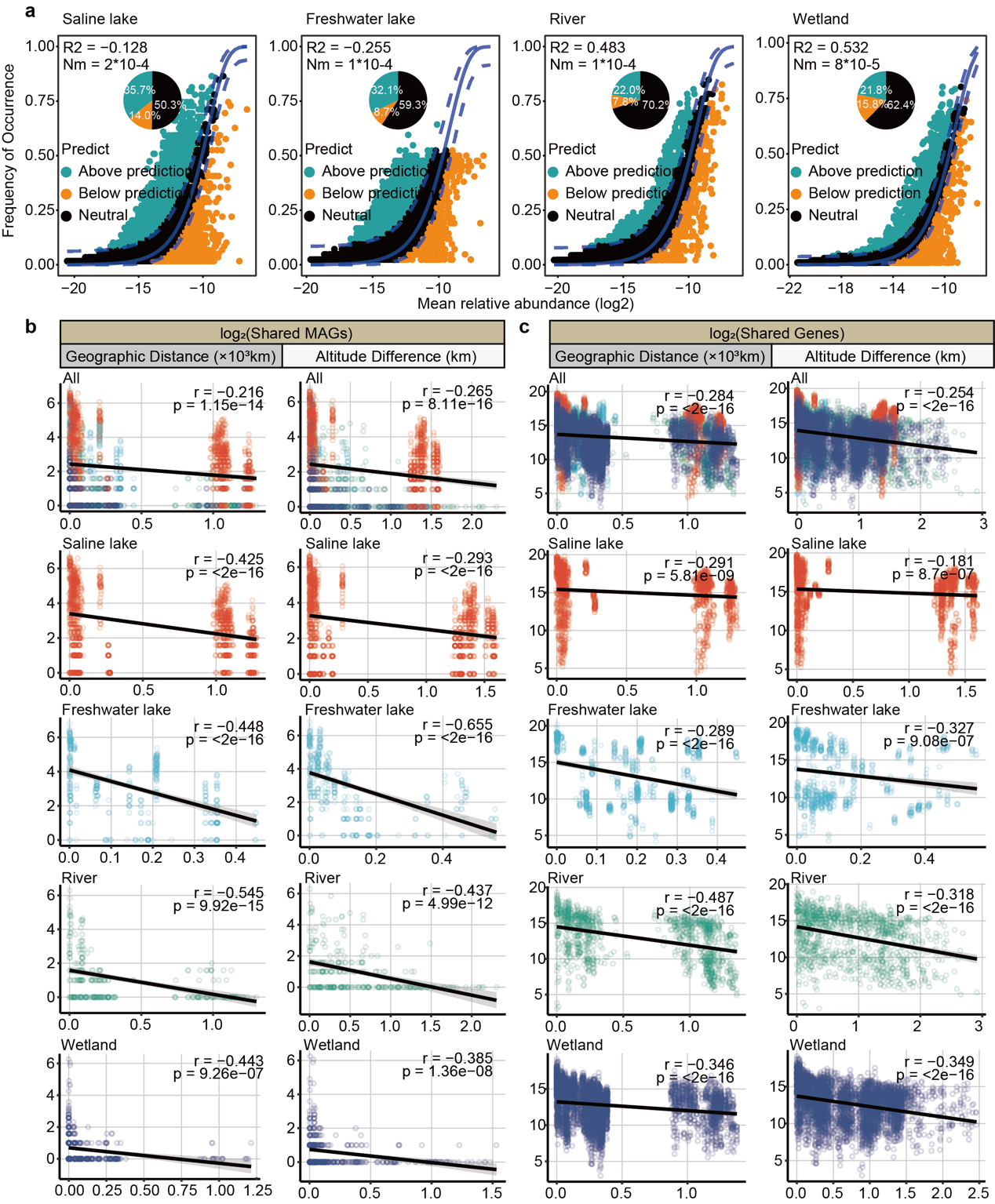


**Figure S9 | Neutral processes and distance-decay relationships in Tibetan Plateau sediment microbes. a** Genome-resolved species occurrence frequencies (Y-axis) are plotted against log10-mean relative abundance (X-axis) to validate the Neutral Community Model (NCM) across ecosystems. Curves show NCM predictions fitted by non-linear least squares (nlsM) using the ‘minpack.lm’ R package. Key parameters include R^2^ (indicating deviation from neutral expectation, with lower values signifying stronger deterministic processes) and Nm (neutrality metric = immigration rate × metacommunity size, where Nm < 1 suggests dispersal limitation). Colored points represent species that occur more frequently than neutral predictions (green) or less frequently (orange), with black points representing the median expectation. **b** Distance-decay relationships (DDR) between the distance (10^3^ km) of samples or the altitude (10 km) of samples and the log2-based number of MAGs shared by samples belonging to the same ecosystems. **c** Distance-decay relationships (DDR) between the distance (10^3^ km) of samples or the altitude (10 km) of samples and the log2-based number of unigenes shared by samples belonging to the same ecosystems. Salmon-generated read counts with TPM normalized are used to calculate shared genes.


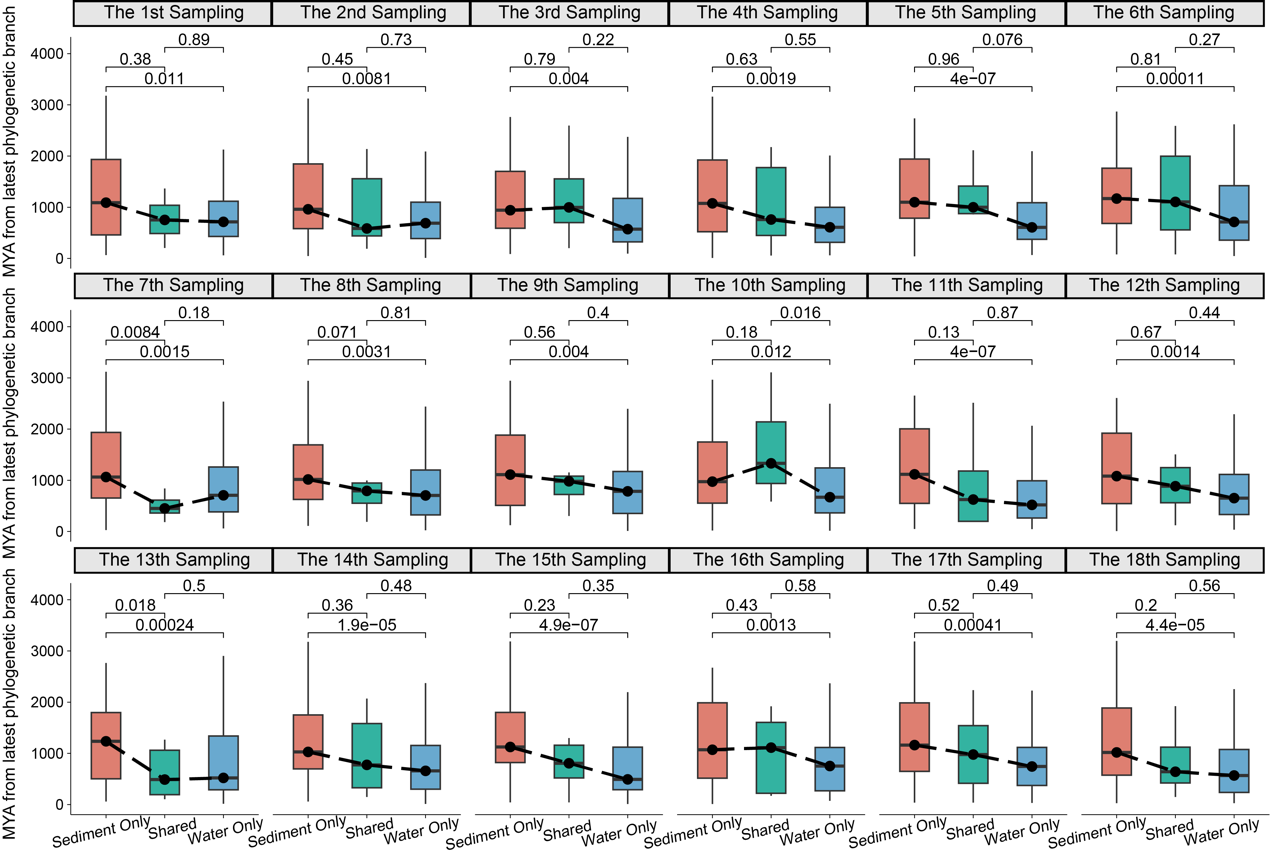


**Figure S10 | The divergence time distribution of microbial lineages derived in the sediment and aquatic samples.** The divergence time (n=3,549) of 18 GTDB-tk based phylogenetic trees was estimated after stratified normal distribution sampling. The boxplots are colored by sites specificity: the red boxplots mean sediment only, the green the red boxplots mean shared between sediment and aquatic samples, and the blue boxplots mean aquatic only. The Y-axis represents the most recent divergence times estimated using RelTime algorithm. Black dots indicate median divergence times per subtree. Intergroup significance is computed via two-sided Wilcoxon rank-sum tests with Bonferroni correction for multiple comparisons.


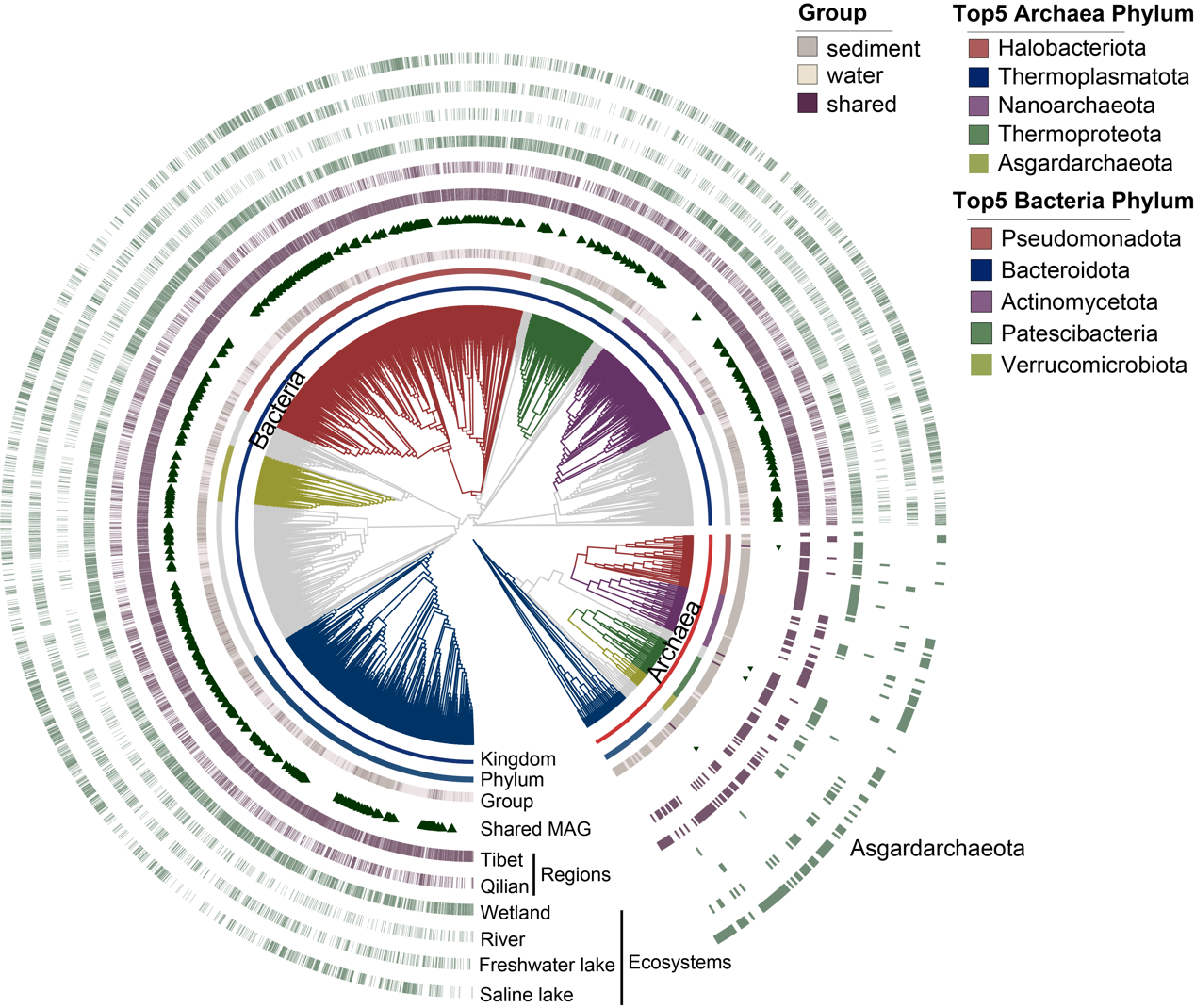


**Figure S11 | Phylogenetic distribution of paired sediment and aquatic samples from Qilian and Tibet across different ecosystems.** Phylogenetic tree of microbial taxa including both Archaea and Bacteria is colored according to the top 5 most diverse phyla in the two categories. The inner concentric rings represent microbial community samples, categorized by their respective group (sediment only, water only, and shared) and region (Qilian and Tibet). The outermost ring is color-coded by ecosystems type (Freshwater Lake, River, Saline Lake, and Wetland). “Paired sediment and aquatic samples” were defined as samples which were obtained from the same sites.

**
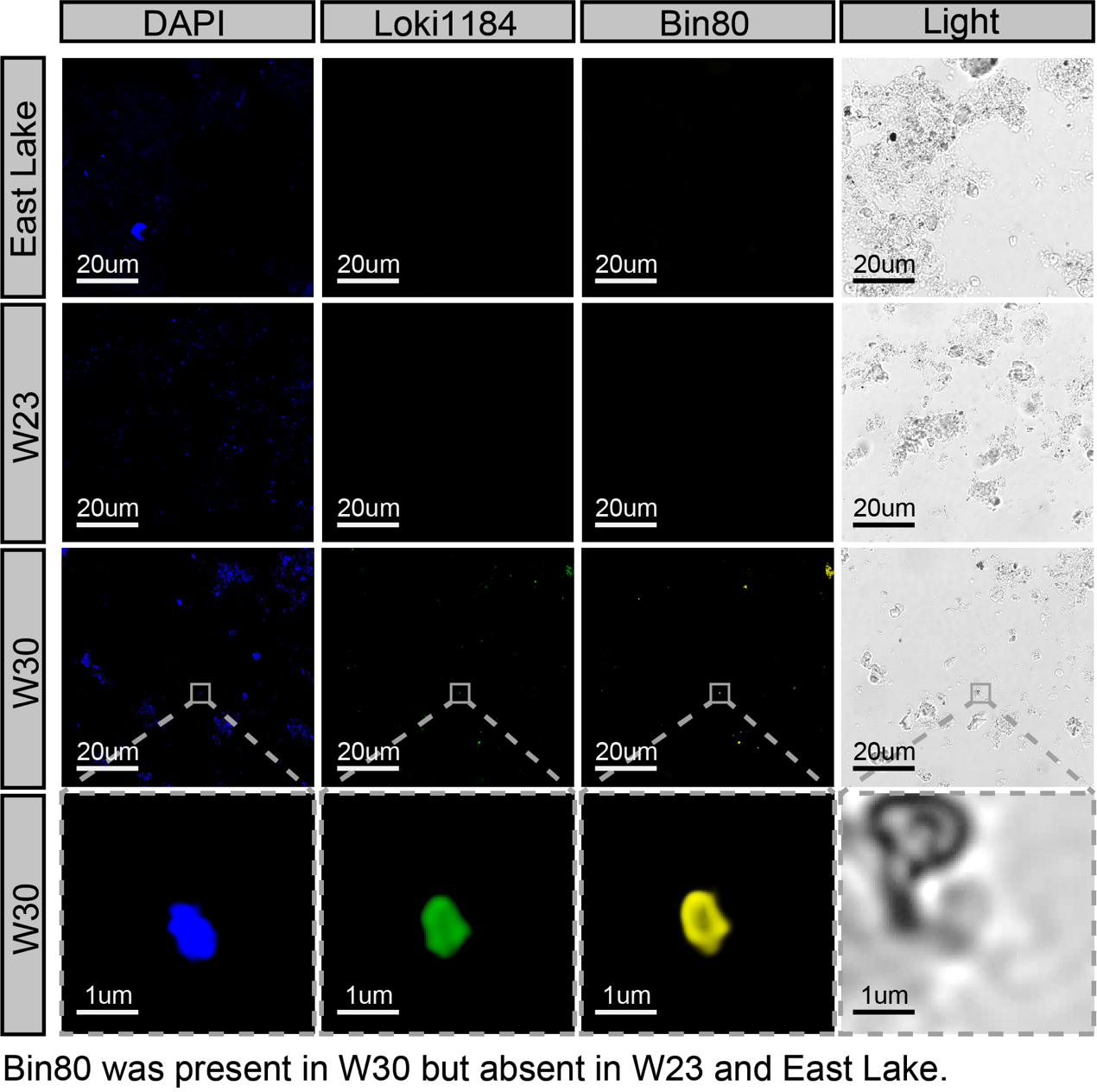
**

**Figure S12 | The Fluorescence in Situ Hybridization (FISH) experiment** **validated the existence of Asgardarchaeota in Tibetan Plateau sediment samples.** Blue, DAPI (4’,6-diamidino-2-phenylindole); Green, Loki1184 (a previously reported probe specific for Lokiarchaeia); Yellow, Bin80 (a custom-designed probe specifically targeting one of the most abundant MAGs). W30, one of the most Asgardarchaeota abundant sediment sample in Qilian; W23, Asgardarchaeota lacking sediment sample in Qilian; East Lake, freshwater sample in East Lake (non–Tibetan Plateau sample).
